# The effects of radiofrequency electromagnetic fields exposure on tinnitus, migraine and non-specific symptoms in the general and working population: A protocol for a systematic review on human observational studies

**DOI:** 10.1016/j.envint.2021.106852

**Published:** 2021-12

**Authors:** Martin Röösli, Stefan Dongus, Hamed Jalilian, Maria Feychting, John Eyers, Ekpereonne Esu, Chioma Moses Oringanje, Martin Meremikwu, Xavier Bosch-Capblanch

**Affiliations:** a Swiss Tropical and Public Health Institute, Socinstrasse 57, CH-4051 Basel, Switzerland; b University of Basel, Petersplatz 1, CH-4003 Basel, Switzerland; c Department of Occupational Health Engineering, Research Center for Environmental Pollutants, Faculty of Health, Qom University of Medical Sciences, Qom, Iran; d Unit of Epidemiology, Institute of Environmental Medicine, Karolinska Institutet, Stockholm, Sweden; e International Initiative for Impact Evaluation, 3ie, c/o LIDC, 20 Bloomsbury Square, London WC1A 2NS, United Kingdom; f Department of Public Health, College of Medical Sciences, University of Calabar, Calabar, Nigeria; g Department of Biology, College of Art & Sciences, Xavier University, Cincinnati, OH, USA; h Faculty of Medicine, College of Medical Sciences, University of Calabar, Calabar, Nigeria

**Keywords:** Microwave, Non-specific symptoms, Sleep, Headache, Tinnitus, Migraine

## Abstract

**Background:**

Applications emitting radiofrequency electromagnetic fields (RF-EMF; 100 kHz to 300 GHz) are widely used for communication (e.g. mobile phones), in medicine (diathermy) and in industry (RF heaters). Concern has been raised that RF-EMF exposure affects health related quality of life, because a part of the population reports to experience a variety of symptoms related to low exposure levels below regulatory limits.

**Objectives:**

To systematically review the effects of longer-term or repeated local and whole human body RF-EMF exposure on the occurrence of symptoms evaluating migraine, tinnitus, headaches, sleep disturbances and composite symptom scores as primary outcomes.

**Methods:**

We will follow the WHO handbook for guideline development. For the development of the systematic review protocol we considered handbook for conducting systematic reviews for health effects evaluations from the National Toxicology Program-Office of Health Assessment and Translation (NTP-OHAT) and COSTER (Recommendations for the conduct of systematic reviews in toxicology and environmental health research).

**Eligibility criteria:**

Peer-reviewed epidemiological studies in the general population or workers aiming to investigate the association between local or whole-body RF-EMF exposure for at least one week and symptoms are eligible for inclusion. Only cohort, case-control and panel studies will be included.

**Information sources:**

We will search the scientific literature databases Medline, Web of Science, PsycInfo, Cochrane Library, Epistemonikos and Embase, using a predefined search strategy. This search will be supplemented by a search in the EMF-Portal and checks of reference lists of relevant papers and reviews.

**Study appraisal and synthesis method:**

Data from included papers will be extracted according to predefined forms. Findings will be summarized in tables, graphical displays and in a narrative synthesis of the available evidence, complemented with meta-analyses. We will separately review effects of local, far field and occupational exposure.

**Risk of bias:**

The internal validity of included studies will be assessed using the NTP-OHAT Risk of Bias Rating Tool for Human and Animal Studies, elaborated to observational RF-EMF studies.

**Evidence appraisal:**

To rate certainty of the evidence, we will use the OHAT GRADE-based approach for epidemiological studies.

**Framework and funding:**

This protocol concerns one of the ten different systematic reviews considered in a larger systematic review of the World Health Organization to assess potential health effects of exposure to RF-EMF in the general and working population.

**Registration:**

PROSPERO CRD42021239432.

## Highlights

• There is public concern to develop non-specific symptoms from EMF.
• No up to date comprehensive systematic review is available.
• Priority outcomes for head exposure are tinnitus, migraine, and headaches.
• Further priority outcomes are sleep disturbances and composite symptom scores.

## 1 Introduction

### 1.1 Background

The technological applications of radiofrequency electromagnetic fields (RF-EMF; frequencies 100 kHz to 300 GHz) have been steadily increasing since the 1950s. RF-EMF are used in medicine (e.g. magnetic resonance imaging, diathermy, radiofrequency ablation), industry (e.g. heating and welding), domestic appliances (e.g. baby monitor, WiFi), security and navigation (e.g. radar and RFID) and especially in telecommunications (e.g. radio and TV broadcasting, mobile telephony). These developments mean that large parts of the global population are now exposed to RF-EMF and more will be exposed in the future. Concern has been raised regarding public health consequences from RF-EMF, in particular related to involuntary exposure from fixed site transmitters, and it is therefore crucial to perform a health risk assessment to support decision-makers and the general public (Hutter et al., 2004, Kheifets and Ritz, 2006, Schreier et al., 2006).

The World Health Organization (WHO) has an ongoing project to assess potential health effects of exposure to RF-EMF. To prioritize potential adverse health outcomes from exposure to these fields, WHO conducted a broad international survey amongst RF experts in 2018 (Verbeek et al., 2021). Six major topics were identified (cancer, adverse reproductive outcomes, cognitive impairment, non-specific symptoms, oxidative stress, and heat-related effects) for which WHO has commissioned systematic reviews to analyse and synthesize the available evidence. In the current paper, we present one of the commissioned protocols for a systematic review on non-specific symptoms in relation to exposure to RF-EMF for human observational epidemiological studies.

### 1.2 Description of the exposure

Radiofrequency electromagnetic fields (RF-EMF) are defined as fields with frequencies from 100 kHz to 300 GHz. Such fields are generated by a large number of equipment both in the general living environment and in workplaces. For these sources, a basic distinction is made between devices operating close to the body, resulting in a near field exposure situation where RF-EMF is coupling to the body, and sources operating far away from the body, which produce a whole-body exposure from a quasi-homogeneous field (ICNIRP, 2020). The differentiation between near and far field depends on several factors, including the dimension of the transmitting antennas. Roughly, far field condition is obtained if the distance between transmitter and receiver is larger than a wavelength. Typical near field sources are mobile phones and Digital Enhanced Cordless Phone (DECT). Typical far field sources include radio- and television masts, mobile phone base stations, DECT base stations, Wireless Local Area Network (WLAN, WiFi) access points or other people's mobile phones. There are many other RF-EMF sources in the everyday environment (e.g. baby monitors, smart meters, avalanche rescue beacons, remote control devices, antitheft devices), in occupational settings (RF polyvinyl chloride welding machines, plasma etching, radar systems) and in medicine (e.g. diathermy, magnet resonance imaging, cardiac pacemakers) (Hareuveny et al., 2015, Mantiply et al., 1997, Vila et al., 2016).

The main variables influencing the interaction of RF-EMF with the human body are the signal frequency (the higher the frequency, the lower the penetration depth), the exposure intensity (defined as the strength of the incident electric and magnetic fields), the exposure duration, the polarization of the field, modulation of the signal and dielectric characteristics of absorbing tissues. The Specific Absorption Rate (SAR in W/kg tissue weight) is the exposure measure of interest and if multiplied by the exposure time, it represents the absorbed RF-EMF whole-body or tissue-specific energy dose. SAR cannot easily be measured inside the human body, and therefore epidemiological studies dealing with whole-body exposure most commonly used external EMF exposure levels such as incident electrical field (V/m) or power flux density (W/m^2^) to quantify exposure levels.

The output power of fixed site transmitters usually is much higher than for devices operating close to the body. However, the electric field strength decreases rapidly with distance (~1/x), which mostly results in relatively low whole-body exposure from environmental sources in contrast to higher but highly localised exposure from devices operating close to the body (Birks et al., 2021, Cabré-Riera et al., 2020, Roser et al., 2017). In a recent dosimetry study taking into account wireless technology use of 1755 adults from four European countries, near field sources contributed on average 69% to the cumulative whole-body dose and 89% to the brain dose (van Wel et al., 2021).

### 1.3 Description of the health outcomes

Some people report several types of non-specific symptoms, which they relate to exposure to RF-EMF. Due to similarities to other forms of idiopathic environmental intolerance (IEI), such as multiple chemical sensitivity, this condition is referred to as IEI attributed to EMF (IEI-EMF) (Rubin et al., 2010, WHO, 2005), although according to a systematic review of identifying criteria the most frequently used descriptive term was “hypersensitive to EMF” (Baliatsas et al., 2012b). The types of reported symptoms vary between individuals. The most commonly reported symptoms are headaches, sleep disturbances and tinnitus, among many others (Baliatsas et al., 2012a, Eltiti et al., 2007, Hillert et al., 1999, Oftedal et al., 2000, Röösli et al., 2004). There is the possibility that different symptoms could result from different types of EMF exposure. However, cluster analyses have not identified that specific symptom clusters are related to specific EMF exposure sources or to EMF exposure in general (Röösli et al., 2004) and the pattern of symptoms is not part of any recognized syndrome (ANSES, 2018).

Prevalence of IEI-EMF was found to vary between countries and years such as 1.5% in Sweden (Hillert et al., 2002), 3.2% in California (Levallois et al., 2002), 3.5% in Austria (Schröttner and Leitgeb, 2008) and in The Netherlands (Baliatsas et al., 2015), 5% in Switzerland (Schreier et al., 2006), about 10% in Germany (Blettner et al., 2009), 13% in Taiwan in 2007 (Meg Tseng et al., 2011) and 4% in Taiwan five years later (Huang et al., 2018). In contrast, the number of people actually seeking medical help for IEI-EMF is substantially lower (Dieudonné, 2020). For instance, in a three-year environmental counselling study in the German part of Switzerland only 70 individuals per year asked for medical advice despite advertising the study to relevant stakeholder groups (Röösli et al., 2011). Some individuals with IEI-EMF report to react to EMF exposure within minutes (Baliatsas et al., 2012a, Baliatsas et al., 2012b, Röösli et al., 2004) but adverse effects may occur only after longer-term exposure or be the consequence of a delayed response. It is also conceivable that RF-EMF causes symptoms but that afflicted persons do not directly attribute them to EMF exposure. Several studies have thus addressed the association between RF-EMF exposure in the everyday environment and occurrence of symptoms in the general population without inquiring individual attribution of causal factors (Auvinen et al., 2019, Baliatsas et al., 2015, Baliatsas et al., 2016, Berg-Beckhoff et al., 2009, Frei et al., 2012, Martens et al., 2017, Mohler et al., 2012, Schoeni et al., 2017, Tettamanti et al., 2020).

Thermal effects of RF-EMF are well understood, and high levels of RF-EMF will result in burns and cause symptoms. Below regulatory limits, thermal effects are minor and cannot cause symptoms (ICNIRP, 2020). If RF-EMF below regulatory limits would cause symptoms, other mechanisms would need to be involved. Physiological effects such as oxidative stress, radical pair mechanisms, or alterations of the human electroencephalogram have been described to occur below regulatory limits but a link to symptoms is not established (Barnes and Greenebaum, 2020, Danker‐Hopfe et al., 2019, Dasdag and Akdag, 2016, Wallace and Selmaoui, 2019).

### 1.4 Rationale for the systematic review

A survey amongst RF experts (Verbeek et al., 2021) ranked “electromagnetic hypersensitivity” as being a topic of high relevance for considering systematic reviews on the grounds of public concerns and the notions of IEI-EMF individuals. Possible immediate effects of RF-EMF exposure on reporting of symptoms have been evaluated in various experimental studies using a blinded, randomised design in a laboratory to apply well-controlled exposure conditions (Schmiedchen et al., 2019). From a practical and ethical point of view, experimental designs cannot be used to study the potential harmful effects of longer-term exposure on delayed or chronic outcomes beyond a few days or weeks. For such effects, observational epidemiological studies are most suitable. In such studies, the occurrence of symptoms in individuals is evaluated in relation to their RF-EMF exposure over a longer time period, irrespective of the individuals’ attribution of symptoms to a specific cause or EMF source, respectively. A number of observational studies have evaluated such longer-term effects, but systematic reviews are scarce and mostly outdated, except for a recent systematic review on tinnitus and mobile phone use (Kacprzyk et al., 2021). Health outcomes other than symptoms are considered in separate systematic reviews organised by the WHO (Verbeek et al., 2021) including a systematic review of non-specific symptoms and RF-EMF evaluated in human experimental studies (Bosch-Capblanch et al., submitted for publication).

## 2 Objectives

The main objective of this systematic review of human observational studies is to provide a comprehensive analysis of the following PECO (Population, Exposure, Comparator, and Outcome) question:

To assess the effects of continuous or repeated local and whole human body RF-EMF exposure per-unit increase (see chapter 4) of one week or longer (E) on the occurrence of non-specific symptoms (O), in the general population or workers (P) and to assess whether there is an exposure–response relationship between these outcomes and RF-EMF exposure levels (C).

Thereby, we will focus on the following five primary hypotheses of RF-EMF effects in the general population:

1. Tinnitus in relation to local exposure of the brain.
2. Migraine in relation to local exposure of the brain.
3. Headaches in relation to local exposure of the brain.
4. Sleep disturbances in relation to RF-EMF from far field exposure sources.
5. Composite symptom scores in relation to whole-body RF-EMF exposure.

The first three hypotheses were set, based on the ground that local exposure of the head from mobile and cordless phone is most pronounced and expected to be most relevant for these outcomes. During sleep, in the absence of own device use, exposure is mostly influenced by far field sources (hypothesis 4). For composite scores, exposure to different body areas may be relevant and thus whole body RF-EMF exposure is expected to be most critical (hypothesis 5). Note that other combinations of the PECO will also be evaluated in an explorative manner according to availability of eligible studies fulfilling the inclusion criteria in terms of outcomes and exposure types.

## 3 Methods

The method of this review is based on the WHO handbook for guideline development (WHO, 2014) complemented by other guidance for systematic reviews of observational studies such as the “Handbook for Conducting a Literature-Based Health Assessment Using OHAT Approach for Systematic Review and Evidence Integration” (NTP, 2019) and COSTER (Recommendations for the conduct of systematic reviews in toxicology and environmental health research) (Whaley et al., 2020).

### 3.1 Eligibility criteria

#### 3.1.1 Types of populations

We will consider studies including participants of the general population (regardless of any restrictions, e.g. in terms of age or gender) as well as studies focusing on workers or persons who attribute their symptoms to EMF exposure (electromagnetic hypersensitive individuals).

#### 3.1.2 Types of exposure

##### 3.1.2.1 Inclusion criteria

Given the public health concerns, it is of interest whether repeated high-level local exposures in the range of 1–2 W/kg under near field conditions (e.g. from a mobile phone) have different effects on health than continuous low-level whole-body exposure under far field conditions.

Studies will be included if they fulfil all three criteria:

(1) The study explicitly declares to evaluate the effects of RF-EMF exposure.
(2) Exposure frequency reported or implied from the source description to be within RF-EMF range as outlined in section 1.2.
(3) Exposure level measured or calculated (dosimetry) by any of the following characteristics:
(i) For local exposure:
  a. The primary choice of exposure for near field sources is time-weighted average or cumulative SAR value of the brain as this represents the RF-EMF dose.
  b. Because SAR measure is rarely available, we will also use other exposure surrogates such as
    i. self-reported or operator-recorded cumulative number of wireless phone calls,
    ii. cumulative duration of calls or time since start of regular wireless phone use,
    iii. or any other well-specified RF-EMF emitting source, for instance in occupational settings.
(ii) For whole-body exposure we will include studies that use:
  a. Time-weighted average or cumulative whole-body SAR value representing daily RF-EMF dose,
  b. whole-body exposure expressed as measured or modelled incident electric field strength (V/m), power density (W/m^2^) or another metric that is convertible to these exposure metrics,
  c. surrogate exposure: studies based on geocoded distance to large broadcast or TV transmitters will be included.
(iii) For occupational sources of exposure:
  a. Time-weighted average or cumulative local or whole-body SAR,
  b. duration of use for local exposure or measured electric field strength or power density for whole-body exposure,
  c. reported as job exposure matrix (JEM) or implied JEM based on occupational titles such as radio or TV transmitter operators, radar workers, TETRA users (e.g. police), RF sealers/welders, dielectric heater operators, short and microwave diathermy operators, and citizens band radio users.

##### 3.1.2.2 Exclusion criteria

We will exclude studies of self-estimated exposure to RF-EMF in general without referring to specific sources such as mobile or cordless phones. A correlation between objective and concurrently collected self-reported data has been demonstrated for mobile phone use (Aydin et al., 2011, Mireku et al., 2018, Schüz and Johansen, 2007, Toledano et al., 2018) and is thus acceptable.

Distance metrics remain challenging as to their interpretation regarding exposure levels. Self-estimated distance to an antenna (Baliatsas et al., 2015) or perceived exposure (Martens et al., 2017) were found not to be correlated to RF-EMF exposure. Geocoded distance to mobile phone base stations had a low correlation with personal RF-EMF exposure (Frei et al., 2010), whereas geocoded distance to radio and TV transmitter was found to be moderate (Spearman: −0.46) (Hauri et al., 2014). Thus, only the latter will be eligible. Self-reported distance to any antenna is not a valid exposure proxy for symptom reporting and may pertain more to perceived exposure rather than to true exposure levels.

In principle, RF-EMF can interfere with implants such as pacemakers or cochlear implants (Sorri et al., 2006) and thus indirectly affect well-being. This interaction is well understood and avoided by proper electromagnetic compatibility testing of implants and is thus not considered in this review.

#### 3.1.3 Types of comparators

We will include studies that have compared at least two different levels of exposure intensity or duration or compare an exposed group to a non-exposed group in the two domains of exposure: local exposure of the brain and whole-body exposure. .

#### 3.1.4 Type of outcome measures

A symptom is a physical or functional alteration that is consciously perceived and experienced as painful, incapacitating, or worrying by a given person. By definition, they can only be assessed through self-reports (or self-reported to a health professional). Symptoms can be non-specific or they can be the consequences of an underlying disease and thus be medically explained. Some outcomes of this review like tinnitus and migraine are well-established diseases, and gold standard for diagnosis is an anamnesis through a health professional based on key criteria and additional examinations. For other symptoms such as headaches or sleep disturbances, it is usually not obvious without in-depth medical examinations whether there exists a medical explanation or whether they are non-specific. To the best of our knowledge, no study in this field of research has attempted to differentiate between medically explained and unexplained symptoms. Thus, these symptoms cannot be read as clinical signs of well-known diseases, but must be interpreted on their own. For this reasons we label them as non-specific. Various standardized scales exist to measure non-specific symptoms. Further, in the research setting, composite symptom scales have been applied, such as the von Zerssen score (von Zerssen 1976) or a scale targeting key symptoms mentioned in the context of IEI-EMF (Eltiti et al., 2007). We will include all symptoms, no matter how serious they are. RF-EMF exposure may act as a trigger for such symptoms, or increase their severity or frequency of occurrence.

We will include any non-specific symptoms as reported by participants of the study and independently whether symptoms were attributed to RF-EMF exposure or not. Actually, attribution of symptoms to a specific source is typically not addressed in epidemiological studies eligible for this review. We consider tinnitus, migraine, headache, sleep quality measures, and composite symptom scores as the main outcomes of this review. Other non-specific symptoms (e.g. fatigue, exhaustion, nervousness) will be included as well.

#### 3.1.5 Types of studies

##### 3.1.5.1 Inclusion criteria

Only observational studies with a longitudinal design will be eligible for inclusion. These are cohort and case-control studies. A cohort study is defined as a study where there are two or more groups exposed to different levels of RF-EMF or no exposure that are followed over time to assess the occurrence of the outcome in question.

Case-control studies depend on identifying cases (so need a diagnostic procedure or otherwise clear case definition). For symptoms with a high prevalence and that vary over time, the case-control study design is not a preferred choice and such studies will not be included. If the outcome occurs rarely and is persistent, which in the scope of this review is the case for tinnitus and migraine, case-control studies are an appropriate design. Therefore, for tinnitus and migraine, we will include cohort and case-control studies. For all other outcomes, we will only consider cohort studies.

##### 3.1.5.2 Exclusion criteria

We will exclude

• cross-sectional studies because there is a lack of temporality in these studies, which makes it difficult to establish causal effects and confounding,
• studies that did not consider any confounder in their analysis,
• studies of patients receiving medical treatment with RF-EMF emitting devices,
• panel studies that study acute and short-term effects only. A panel study is a special case of a cohort study that typically includes more frequent follow-up measurements (e.g. using a symptom diary) and thus considers mostly effects occurring within a relatively short time of a few hours to a few days. For such acute effects, observational studies are suboptimal as they cannot control blinding of exposure and thus may be vulnerable to well established nocebo effects (Bräscher et al., 2017, Schmiedchen et al., 2019, Van den Bergh et al., 2017).

A special case are field trials. Similar to observational studies, such studies are done in the everyday environment of study participants. However, if they follow an experimental approach, e.g. by turning on and off a mobile phone base station (Danker-Hopfe et al., 2010), such studies will qualify for a review on human experimental studies (Bosch-Capblanch et al., submitted for publication).

##### 3.1.5.3 Years considered

Any year of publication that is recorded in the scientific databases will be considered.

##### 3.1.5.4 Publication language

We will include studies written in any language. Articles in languages other than the ones spoken by the reviewers (English, German, Spanish, Catalan, French and Portuguese) will be discussed with collaborators in the network of authors’ institutions proficient in those languages. However, considering that title and abstract of non-English articles published in peer-reviewed journals are in English, only English terms will be used to search the publication databases.

##### 3.1.5.5 Publication types

We will include studies reported as peer-reviewed publications.

#### 3.1.6 Types of effect measures

For dichotomous outcomes, we will use the Relative Risk (RR) as the measure of the effect. We will also consider Odds Ratios (OR) and Hazard Ratios (HR). Because the incidence of the symptoms of interest is not always low, we will transform all effect sizes into RRs (Grant, 2014). For continuous outcomes, we will use mean differences as the effect size. When the same symptom is measured with different scales, we will use standardised mean differences as the effect size.

Effect measures of analyses based on exposure categories will be expressed per unit increase of corresponding exposure measures.

### 3.2 Information source and search strategy

Eligible studies will be identified by literature searches in the databases Medline, Web of Science, PsycInfo, Cochrane Library, Epistemonikos and Embase. Each database strategy will be tailored to the characteristics of each platform together with encompassing its controlled language (index) features, where appropriate, and using a combination of title, abstract and author keywords. The strategy will use two study design filters – observational and experimental studies for Bosch-Capblanch et al. (submitted for publication) – as outlined above in the inclusion criteria of study types, with results given for each. We will also consult the EMF-Portal, a dedicated database of the scientific literature on the health effects of exposure to electromagnetic fields (https://www.emf-portal.org/en). These searches will be supplemented by checks of the reference lists of previous systematic reviews, as far as such reviews are available. The software Endnote will be used to manage the bibliography.

Based on the inclusion criteria, we have developed a search strategy separately for the databases to be searched (see sample Medline and Web of Science strategies in Appendix A). To obtain a vigilance balance between sensitivity and specificity the search strategy combines the three elements (i) different terms describing RF-EMF exposure, (ii) different terms for relevant study designs, (iii) different terms for the outcome of interest. We will start our search from the first available year in the respective database.

### 3.3 Study selection

First, the relevance of the identified papers will be checked based on titles and abstracts, conducted by two reviewers. At this stage, we will exclude records that are not relevant and certainly will not fulfil one or more of the inclusion criteria listed above. This will result in a list of references for which again two reviewers will independently assess inclusion based on the full-text of the article. Studies excluded in this step will be listed in a supplementary file of the review paper including reasons for exclusion. Cross-sectional studies that fulfil the inclusion criteria except the longitudinal design criteria, will be hallmarked in this list. This step will result in a list of included articles.

If findings from a study are described in more than one article, we will consider all these papers as one study only. The third step will result in a list of included studies. In all steps, any disagreement between the two reviewers will be resolved by discussion. If no consensus can be reached, a third reviewer will be consulted. We will document the selection process in a study flow diagram according to the PRISMA reporting guidelines (Liberati et al., 2009).

### 3.4 Data extraction

For each study included in the current review, a standard set of details will be extracted from the relevant publication(s). This includes bibliographic information including description of the study methods and the study sample (Appendix B), risk of bias instructions (Appendix C), and study results for later result synthesis (sheet 3_Outcomes in Appendix B).

Based on Excel forms (Appendix B), two reviewers will work independently to extract quantitative and other key data. Possible disagreements between reviewers will be resolved by discussion including a third reviewer. If one of the authors of the review is also an author of an included study, we will make sure that this author will not extract data from their own study and will not judge the risk of bias.

In terms of exposure–response data, we will extract all information that is provided in a paper for corresponding syntheses of the results. This may include duration and frequency of use or categorical and linear exposure response analysis results. We will extract effect estimates based on the most comprehensive confounding adjustment.

If there is more than one article per study, we will use the original paper (i.e., the first publication), while findings reported in subsequent articles based on the same individual data will only be extracted if relevant or if comprising a more comprehensive sample or address a type of eligible population, exposure or outcome not addressed in the original paper. In this case, Appendix B will be filled in for each paper. The same holds for pooled analysis combining original data from a set of primary studies. Pooled analyses of primary studies are eligible for inclusion in the current systematic review if they include a more comprehensive set of data than previously published in individual primary studies.

*Dealing with missing data*

If data necessary for the analysis are missing from the articles, we will ask the corresponding author for additional information. In case of no response, we will ask first, last and co-authors with reminders, if necessary, or we will endeavour to calculate these from other data available in the papers when feasible.

### 3.5 Risk of bias assessment

For evaluating the internal validity, we will conduct a risk-of-bias assessment using the “Risk of Bias Rating Tool for Human and Animal Studies” developed by the NTP Office of Health Assessment and Translation (OHAT) (NTP, 2015, Rooney et al., 2014), which was modified for the specific exposure and outcomes considered in this review (Appendix C). We only considered domains relevant for cohort and case-control studies as suggested by OHAT. In the instruction form, all instructions from the original OHAT document (NTP, 2015) are printed in black. All elaborations to the form, which were informed by topic knowledge of the review team, discussions with other WHO review teams, ROBINS-I (Sterne et al., 2016) and COSTER (Whaley et al., 2020) are printed in blue for easier recognition. Studies will be assessed across six domains with eight different questions, with detailed criteria elaborated for each domain in the “risk-of-bias instruction” document (Appendix C). The following eight questions are considered: Selection/participation bias, confounding, attrition/exclusion bias, exposure assessment errors, outcome assessment errors, selective reporting, and other biases, which includes the two sub-questions related appropriate statistical methods, and reverse causality. Reverse causality may occur if IEI-EMF individuals take measures to reduce their RF-EMF exposure when developing symptoms (Röösli et al., 2010). If not adequately considered in the longitudinal design, this would downward bias the effect estimates towards a false protective effect of RF-EMF, because change of symptoms score would be negatively correlated with exposure status (Appendix C).

Using the instruction guide (Appendix C), risk of bias will be evaluated in the “risk of bias form” (sheet 2c_RoB, Appendix B) for each paper separately and for each type of outcome, each type of exposure, each type of exposure assessment method and type of population, if applicable. Biases such as confounding may differ according to these three aspects. As proposed by the OHAT Risk of Bias tool, the answer format is definitely low risk of bias (++), probably low risk of bias (+), probably high risk of bias (− or not reported “NR”), or definitely high risk of bias (−−). For each study result that is considered to be at probably or definitively high risk of bias, the reviewer will also judge the direction of the bias (or combined biases) for the corresponding effect estimate. This includes the following four answer formats: false positive risk (i.e. overestimation of harmful effect), bias towards absence of an association (underestimation of harmful effect), false protective finding (i.e. favours beneficial effect) and unpredictable.

It has to be emphasised that effects on symptoms from mobile phones and other electronic communication media can be unrelated to EMF exposure. This includes sleep deprivation from incoming calls and text messages during night (Foerster et al., 2019) or psychological and somatic arousal through media content (Cain and Gradisar 2010). Further, it has been postulated that electronic media use may result in less physical activity (Edelson et al., 2016), higher night time eating (Cha et al., 2018), higher BMI (Fatima et al., 2015), or media addiction (Roser et al., 2016, Samaha and Hawi, 2016). In the risk of bias analysis, we will evaluate whether most relevant confounders have been considered (see Appendix C). Importantly, some studies have developed further specific strategies to deal with this type of confounding and to differentiate between associations related to usage and associations related to RF-EMF dose. Mobile phones and to some extent also other devices have an efficient power control (Gati et al., 2009, Persson et al., 2012, Popović et al., 2019). Depending on the network settings, signal quality and the type of usage, output power of mobile phones can vary with a factor of one million (Mazloum et al., 2019). Some studies have used such information and considered the average output power of mobile phone calls in the GSM and UMTS network, to achieve an exposure surrogate, which better represents EMF dose than just usage (Auvinen et al., 2019). Other studies used negative exposure control variables such as number of text messages, which implies virtually no RF-EMF exposure, to compare associations of different usage proxies (Schoeni et al., 2017). We will extract such information in the risk of bias including the impact on the study results.

#### 3.5.1 Key criteria for 3-Tier system

As suggested by the OHAT handbook, we apply a 3-Tier system for later synthesizing study findings when risks of bias vary across studies or across different analyses from the same study. The tiering approach is based on the following three key criteria:

(1) Did the study design or analysis account for important confounding and modifying variables?
(2) Can we be confident in the exposure characterization?
(3) Can we be confident in the outcome assessment?

We will produce heat maps for visualization of the risk of bias. Note that within the same study the result of the tiering approach may vary depending on the type of outcome, type of exposure and type of exposure assessment method considered.

A Tier 1 study result must be rated as “definitely low” or “probably low” risk of bias for all three key elements mentioned above AND have no other critical bias identified. For near field exposure studies Tier 1 studies need to have applied any kind of analytic strategy to differentiate between device usage and RF-EMF exposure as outlined in chapter 3.5. A Tier 3 study result must be rated as “definitely high” or “probably high” risk of bias for key elements. A Tier 2 study result meets neither the criteria for 1st or 3rd Tiers (NTP, 2019).

Funding source and disclosure of conflict of interest is not a specific domain in the OHAT Risk of Bias tool, but we will collect such information during data extraction. Funding source is recommended as a factor to consider when evaluating risk of bias of individual studies for selective reporting, and then again for evaluating the body of evidence for publication bias. Funding source should be considered as a potential factor to explain apparent inconsistency within a body of evidence. Based on empirical evidence (Huss et al., 2007) we may consider bias in both directions, downplaying associations because of industry bias or highlighting associations to attract research funding, for instance in unfunded pilot studies.

## 4 Synthesis of results

Findings will be summarized in tables, graphical displays and in a narrative synthesis of the available evidence. For the five primary hypotheses of this review, we will conduct a random-effects meta-analysis for all eligible studies in STATA. For other combinations of the PECO a meta-analysis will be conducted if sufficient studies are available, which are comparable in terms of exposure source and type of outcome. Meta-analyses of RRs or changes in symptom score will be performed according to the original studies. We will assign a single exposure value to each category. For closed categories, the geometric mean of the upper and lower bounds of the exposure categories will be used; for the (uppermost and lowest) open-ended categories, we will assign an estimated median value as proposed by (Il’yasova et al., 2005). Exposure-response trends will be evaluated by means of meta-regression.

In each meta-analysis, we will not combine results from completely or partially overlapping populations. We will also not combine in a pooled estimate multiple data from the same subject obtained by different exposure or outcome assessment methods. We will conduct separate analyses for local exposure of the brain and for whole-body exposure. Occupational settings may involve near field, far field or a mixture of both, depending on the job (Vila et al., 2016). Occupational exposure will be considered either as near field exposure of the brain, or as whole-body exposure. If the occupation setting is too complex to make such a decision, it will be analysed separately.

Based on an initial screening of the literature, we will consider duration of wireless phone use as the primary exposure metric for local RF-EMF exposure to the brain. We will express changes in risk or score changes per 100 min call duration per week. For whole-body exposure, V/m will be the primary exposure metric. In case of different exposure metrics, we will do recalculation based on standard calculations (e.g. for V/m, A/m and W/m^2^) or use most recent transfer functions for transferring cumulative brain SAR values into duration of mobile phone use or vice versa and whole-body SAR to an electric field strength (van Wel et al., 2021).

*Subgroup analyses and assessment of heterogeneity*

We will evaluate heterogeneity of the findings according to the PECOs elements and quantify the statistical heterogeneity between studies with the tau-square measure and calculate 80% prediction intervals (IntHout et al., 2016), where the number of studies for various subgroup permits. We will group according to outcomes and according to the types of populations (adults, children, adolescents, EHS, or workers). For studies addressing local exposure of the head, we will conduct separate evaluations for mobile and cordless phones. For whole-body exposure, separate evaluations will include total and a restriction to far-field exposure (i. e mobile phone base stations, broadcast transmitters, WiFi access points). If study availability permits, we will also do separate analysis for various types of far field exposure varying in terms of frequency and modulation.

In the synthesis, we will focus on consistency across various subtypes of exposures (mobile vs. cordless phone or whole-body and far field exposure levels), different exposure assessment methods (self-reported, database/operator, measurements, modelling, mixed), data analysis approaches (exposure at baseline [cohort], cumulative exposure between baseline and follow-up or change in exposure between baseline and follow-up), and type of exposure–response analysis (categorical, linear). In case results are heterogeneous for different subtypes of exposure, we will evaluate whether this indicates bias or indicates that effects depend on detailed characteristics of exposure such as frequency, duration, modulation or body localisation.

To inform the quality of evidence assessment and as suggested by the OHAT handbook, we will also group according to the risk of bias tiering by restricting primary analysis to studies with lower risk of bias and perform a sensitivity analysis to evaluate potential changes in conclusions if studies at higher risk of bias were included. We will conduct a sensitivity analysis to assess the effect of assumptions made during the review process on the conclusions.

## 5 Assessing certainty in the body of evidence

The certainty rating for each set of PECO considered sufficiently similar to be combined is done according to the procedure of the OHAT handbook (NTP 2019), which is based primarily on guidance from GRADE (Guyatt et al., 2008). The GRADE approach was originally developed to rate the body of evidence in the field of clinical medicine. In a first step, the quality of a body of evidence is rated based on the initial certainty from the key figures of available study designs. Randomised control trials (RCT) are practically and ethically not applicable for long-term effects of RF-EMF as discussed for other environmental risk factors (Morgan et al., 2016). Thus, the study design most applicable and available for the specific topic of the review is used as a starting point, as also has been done for the development of the WHO noise guidelines (WHO, 2018). These are prospective cohort studies and for rare outcomes case-control studies. Since some extent of bias cannot be excluded by design in observational research of RF-EMF effects on symptoms, we will give these studies a moderate (score 3) initial certainty rating. Based on this point of departure, the evidence base will be rated down or up whenever one or more of the criteria for downgrading or upgrading (described above) were met.

The following five factors are used for downgrading the quality of the body of evidence from observational studies by one or two levels for each set of PECO. Arguments will be documented according to the template of Appendix D.

1. Risk of bias across studies for each outcome (not likely, serious, very serious):

For rating the risk of bias across studies, a heat map (visual summary of the risk of bias) is prepared for each outcome as proposed by the OHAT handbook. This highlights the general strengths and weaknesses of all included studies and highlights particular risk of bias domains that could be explored when evaluating inconsistencies between studies. In this process, we will also consider the direction of bias and magnitude of effect. Risk of bias can lead to downgrading with one or two levels according to the seriousness of bias. Judgement will be based on the number of studies, their impact on the meta-analysis and the seriousness of the risk of bias in these studies. One small study with a very serious risk of bias but hardly an influence on the results synthesis will not be a reason to downgrade. However, risk of bias in studies with considerable weight in the result synthesis will be taken into account. We will use the tiering approach described above to guide the decision to downgrade. No downgrading will be conducted if most information is from Tier 1 studies with low risk of bias for all key domains. Downgrading by one unit (serious risk of bias) is done if most information is from Tier 1 and Tier 2 studies. Downgrading by two units (very serious risk of bias) is done if the proportion of information from Tier 3 studies at high risk of bias for all key domains is sufficient to affect the interpretation of results.

2. Inconsistency of results between studies (none, serious):

Inconsistency between studies means that there is a considerable difference in effect size between studies, for example, if there are studies in the body of evidence that show a preventive effect and other studies that show a harmful effect. It is important to evaluate if any observed heterogeneity is due to specific differences between studies by means of subgroup analyses such as comparing studies with mainly adults with those with mainly children. If heterogeneity can be explained, there is no reason for concern.

A common measure of heterogeneity is the I^2^ statistic. Because the I^2^ statistic is a relative measure, it is difficult to make a judgement of the absolute amount of heterogeneity. Therefore, we will use the prediction interval (PI) estimated from the underlying distribution of effect estimates (IntHout et al., 2016).

To make a judgement about the amount of heterogeneity that would be a reason for concern and a reason to downgrade if it cannot be explained, the following approach will be followed. If the 80% PI overlaps with the null value (RR = 1), it means that studies show both beneficial and harmful effects of exposure. If the 80% PI for a specific meta-analysis of RRs is of the same size as the 95% confidence interval of the mean (pooled) effect estimate, it indicates that there is no more variation in effect sizes than the statistical uncertainty. Then there is no reason for concern about heterogeneity. However, if the PI is considerably wider than the confidence interval (for example double the size) and overlaps with 1, then there is reason for concern about heterogeneity. The effect sizes of the studies vary so much that with different samples of studies the conclusions of the meta-analysis could be substantially different apart from statistical uncertainty. In this case, we will downgrade the certainty of the body of evidence by one level. PI will be calculated per outcome and type of exposure. In case there is concern about inconsistency, it will be evaluated whether this can be applied by factors outlined in the heterogeneity analysis. Evidence will be downgraded if heterogeneity cannot be explained. We would also downgrade if results for comparable exposure sources (e.g. mobile vs. cordless phones or mobile phone base stations vs. other far field exposure sources) are not consistent, when taking into account the level of exposure.

The more studies available for a meta-analysis on a specific outcome and type of exposure, the better the PI can be estimated. Thus, there is concern if evidence is generated from few studies only and PI cannot be estimated. If only two or less exposure–response results are available for a specific outcome and type of exposure, certainty of evidence will be downgraded by one item as confidence in the evidence quality is low when the study base is thin.

3. Indirectness of evidence in the studies (none, serious):

This item refers to the extent to which PECO in the studies of the systematic review reflects the original PECO that was formulated at the start of the systematic review process (chapter 3.1.1 to 3.1.4). If there are considerable differences between the characteristics of those exposed to electromagnetic fields in the real world and the characteristics of those evaluated in the studies, we will downgrade the quality of the evidence by one level. This would, for example, be the case if the evidence would be based on studies of exposure very different from the exposure in the general population, if the exposed population is a very specific occupational group or if the exposure duration is very different than in the everyday environment.

4. Imprecision (none, serious):

As most of the outcomes under study are common, even small cohorts can provide relatively precise risk estimates, in particular for common exposure situations. However, in the absence of a biological mechanism, most observational studies did not follow a hypothesis-driven approach but rather an explorative strategy addressing many symptoms within the same study. On the one hand, this increases the likelihood of a false positive (type 1 error) result in a given study, in particular for small studies. This may produce an overestimation of the effect size for the population, although in a meta-analysis including also large studies, the effect may be small. On the other hand, researchers apply procedures to correct for such chance findings, such as Bonferroni correction. However, such corrections may increase the likelihood of a false negative (type 2 error) if the sample size is not very large and should be considered when evaluating the evidence. Adequate power is especially important when interpreting findings that do not provide support for an association. OHAT uses 95% confidence intervals as the primary method to assess imprecision. We will downgrade the evidence if the upper limit of the confidence interval of a relative risk is >2 in a non-significant effect estimate. For a significant effect estimate, downgrading is done if the upper limit of the confidence estimate divided by the point estimate is >1.5. An analogue rule will be applied to the logarithm of beta coefficients referring to score changes.

The OHAT handbook mentions the difficulties to distinguish between wide confidence intervals due to inconsistency and those due to imprecision. As suggested by OHAT we will prevent from downgrading twice unless if studies are both very inconsistent and imprecise.

5. Publication bias detected in a body of evidence (none, serious):

Reporting bias or publication bias occurs when the publication of studies depends on the nature and direction of the results, so that the results in published studies may be systematically different from those in unpublished studies. Publication bias can thus lead to under- or overestimation of the effect of RF-EMF due to selective publication of studies. According to the OHAT handbook, some degree of publication bias is likely on any topic; however, downgrading is reserved for cases where the concern is serious enough to significantly reduce certainty in the body of evidence.

Where enough studies are available per outcome (n ≥ 10), we will conduct a visual inspection of the study results in relation to study size and standard error of effect estimates. Thereby, we will also consider the chronology of the studies, to see whether early studies were more likely to report associations. We will also conduct standard meta-analytic tests of publication bias (e.g. Egger's test). We will downgrade the quality in case we suspect publication bias based on such tests for publication bias. Where <10 study estimates contributed to the evidence base, we will compare study findings, according to study size and publication year as early positive studies, particularly if small in size, are suspect. In subgroup analysis, we will also evaluate findings by funding source as publication bias can be suspected for small studies sponsored by industries, non-government organizations (NGOs), or authors with conflicts of interest (Guyatt et al., 2011). In case we find substantial evidence for publication bias, we will downgrade the certainty in the evidence quality by one unit.

The following three factors are used for upgrading the certainty in the quality of evidence of observational studies (Appendix D):

1. Large magnitude of effect (small, large, very large):

The GRADE working group proposes to upgrade the certainty of the evidence in observational studies if the pooled-effect size is large or very large, so that 'the study design that is more prone to bias is unlikely to explain all of the apparent benefit (or harm)'. The cut-off points for a large effect size for harm proposed by GRADE are RR > 2 or very large RR > 5. We consider these effect magnitudes to be appropriate for the topic under review and will rate a pooled relative risk of > 2 or < 0.5 as of high magnitude and > 5 or < 0.2 as of very high magnitude and would upgrade the certainty of the evidence quality by one or two units, respectively. For score changes, we consider an effect, which is >50% of the standard deviation as of high magnitude (very high magnitude: two times the standard deviation). It is important to realise that if the RRs are incremental, i.e. indicate an increase per unit of exposure; these have to be converted to a realistic exposure contrast. This will be informed by the interquartile range of most recent studies.

2. Exposure Response gradient (no, yes):

OHAT will upgrade for evidence of a monotonic exposure–response gradient and for evidence of a non-monotonic dose response when data fit the expected pattern according to prior knowledge. For the topic of our review, there is no expectation of a non-monotonic exposure–response pattern. This aspect will be evaluated across studies using the same type of exposure. Exposure-response gradient is considered to be consistent, if a test for trends across exposure categories is found to be significant. Depending on the original data, number of categories should be between four and six when performing a test for trend. When evaluating the exposure–response gradient we may also consider other aspects of exposure than intensity such as exposure duration.

3. Residual confounding (towards null, not likely):

Another proposed reason for upgrading is if all plausible confounding would shift the RR towards the null and still there would be a significant RR. This requires considerable judgement of possible confounders. In most RF-EMF studies on symptoms, there would be a long list of possible confounders and effect modifiers that would shift the RR in both directions, which cannot be defined with certainty given the lack of established biological understanding. However, if it can be reasonably argued that most relevant confounding would have reduced the observed RR towards 1 (e.g. healthy worker effects in occupational studies), then this will be a reason to upgrade the certainty of the evidence with one unit.

The effect of residual confounding can be indirectly assessed in studies on local exposure that attempted to differentiate between usage and RF-EMF dose as described above in chapter 3.5. If the conclusions from these studies support RF-EMF as an explanation for observed associations, this would increase our certainty in an observed association by one unit (opposite procedure as explained above when downgrading due to risk of bias).

The OHAT handbook suggests evaluating consistency across animal studies, across dissimilar populations and across study types. This is beyond the scope of this systematic review and will be conducted at a later stage by the WHO task force.

## 6 Reporting

We will use the PRISMA guidelines for the reporting of systematic reviews to report the findings of our review (Liberati et al., 2009).

## 7 Financial support

This project is funded by the World Health Organization (RAD 2020/1048990-0; EHC-RAD 2020/994772-0) and by intramural funds of the Swiss Tropical and Public Health Institute.

## Role of funders

A strict oversight was exercised by the WHO Secretariat to ensure that all commissioned systematic reviews were planned according to a harmonized and good practice standard.

## Conflicts of interest

Martin Röösli’s research is entirely funded by public or not-for-profit foundations. He has served as advisor to a number of national and international public advisory and research steering groups concerning the potential health effects of exposure to nonionizing radiation, including the World Health Organization, the International Agency for Research on Cancer, the International Commission on Non-Ionizing Radiation Protection, the Swiss Government (member of the working group "mobile phone and radiation" and chair of the expert group BERENIS), the German Radiation Protection Commission (member of the committee Non-ionizing Radiation (A6) and member of the working group 5G (A630)) and the Independent Expert Group of the Swedish Radiation Safety Authority. From 2011 to 2018, M.R. was an unpaid member of the foundation board of the Swiss Research Foundation for Electricity and Mobile Communication, a non-profit research foundation at ETH Zurich. Neither industry nor nongovernmental organizations are represented on the scientific board of the foundation.

Maria Feychting has a permanent position as Professor of Epidemiology at Karolinska Institutet, Stockholm Sweden since 2005. She has served as advisor to a number of national and international public advisory and research steering groups concerning the potential health effects of exposure to non-ionizing radiation, including the WHO (ongoing), Public Health England Advisory Group on Non-ionising Radiation - AGNIR (2009-17), the Norwegian Public Health Institute (2010-12), the Swedish Council for Working Life and Social Research (2003-2012), Swedish Radiation Safety Authority’s independent scientific expert group on electromagnetic fields (2003-11). She was member of the International Commission on Non-Ionizing Radiation Protection (ICNIRP), an independent body setting guidelines for non-ionizing radiation protection (2008-May 2020), and vice chairman of the Commission May 2016-May 2020.

## CRediT authorship contribution statement

**Martin Röösli:** Conceptualization, Funding acquisition, Investigation, Methodology, Project administration, Resources, Supervision, Writing – original draft, Writing – review & editing. **Stefan Dongus:** Investigation, Methodology, Writing – review & editing. **Hamed Jalilian:** Investigation, Methodology, Writing – review & editing. **Maria Feychting:** Methodology, Writing – review & editing. **John Eyers:** Methodology, Resources, Writing – review & editing. **Ekpereonne Esu:** Methodology, Writing – review & editing. **Chioma Moses Oringanje:** Methodology, Writing – review & editing. **Martin Meremikwu:** Methodology, Writing – review & editing. **Xavier Bosch-Capblanch:** Conceptualization, Funding acquisition, Methodology, Resources, Writing – review & editing.

## Declaration of Competing Interest

The authors declare that they have no known competing financial interests or personal relationships that could have appeared to influence the work reported in this paper.
