# Supplementary material for: The effects of radiofrequency electromagnetic fields exposure on tinnitus, migraine and non-specific symptoms in the general and working population: A protocol for a systematic review on human observational studies
Source: Environ Int. 2021 Dec;157:106852. doi: 10.1016/j.envint.2021.106852 (PMC8484767; doi:10.1016/j.envint.2021.106852)
Supplement: Supplementary data 1 [file mmc1.pdf]

1. **Web of Science (Science Citation Index & Social Sciences Citation Index) – Searched 4th February 2021**

28 **3,092** [Amalgamated Results – Observational/Experimental Designs – Limited by Web of Science Subject Categories]

#24 NOT #25
Refined by: WEB OF SCIENCE CATEGORIES: (NEUROSCIENCES OR PSYCHOLOGY BIOLOGICAL OR CLINICAL NEUROLOGY OR SOCIAL SCIENCES INTERDISCIPLINARY OR PUBLIC ENVIRONMENTAL OCCUPATIONAL HEALTH OR PSYCHOLOGY APPLIED OR BEHAVIORAL SCIENCES OR PSYCHIATRY OR MEDICINE RESEARCH EXPERIMENTAL OR AUDIOLOGY SPEECH LANGUAGE PATHOLOGY OR BIOPHYSICS OR ENVIRONMENTAL SCIENCES OR PSYCHOLOGY OR PSYCHOLOGY MULTIDISCIPLINARY OR PSYCHOLOGY CLINICAL OR PSYCHOLOGY EXPERIMENTAL) AND **[excluding] DOCUMENT TYPES: (MEETING ABSTRACT OR PROCEEDINGS PAPER)**
Indexes=SCI-EXPANDED, SSCI Timespan=1970-2021

27 3,202

#24 NOT #25
Refined by: WEB OF SCIENCE CATEGORIES: (NEUROSCIENCES OR PSYCHOLOGY BIOLOGICAL OR CLINICAL NEUROLOGY OR SOCIAL SCIENCES INTERDISCIPLINARY OR PUBLIC ENVIRONMENTAL OCCUPATIONAL HEALTH OR PSYCHOLOGY APPLIED OR BEHAVIORAL SCIENCES OR PSYCHIATRY OR MEDICINE RESEARCH EXPERIMENTAL OR AUDIOLOGY SPEECH LANGUAGE PATHOLOGY OR BIOPHYSICS OR ENVIRONMENTAL SCIENCES OR PSYCHOLOGY OR PSYCHOLOGY MULTIDISCIPLINARY OR PSYCHOLOGY CLINICAL OR PSYCHOLOGY EXPERIMENTAL)

26 7,892

#24 NOT #25

25 218,288

TS=((messag* or text or texts or texting or sms or app) NEAR/5 (treat* or therap* or cessation) or mhealth or m-health or "mobile health" or ablation* or transcranial* or trans-cranial*)

24 10,295

#23 OR #22

23 8,018

#21 AND #20 AND #14

22 4,714

#21 AND #20 AND #9

21 2,388,535

TS=(electrohypersensitiv* or IEI-EMF or "environmental intolerance" or electrosensitiv* or "electric* sensitivity" or sleep* or memory or hearing or auditory or self-report* or "self

report*" or dizziness or dizzy or fatigue or tiredness or tinnitus or headache* or nocebo or psychophysiologic* or psychosomatic or psycho-somatic or hypersensitiv* or well-being or "well being" or "quality of life" or HRqol or (concentrat* adj2 (abilit* or inability or capacit*)) or nervous or neurasthen* or vegetative or ((body or bodily or physical or physiological* or unspecifi* or non-specifi* or indetermin* or undetermin*) NEAR/3 (sensation* or feeling* or symptom* or change*)))

20 1,084,199

#19 OR #18 OR #17 OR #16 OR #15

19 818,722

TS=(electromagnetic or electro-magnetic or radiofrequency or radio-frequency or DECT or "cordless telecomm*" or ((radio or telephon* or mobile or base or radar or broadcast* or television or tv) NEAR/2 (station* or mast or masts or transmitter* or antenna*)) or TETRA or "plastic sealer*" or wifi or wi-fi or wlan or "wireless area network*" or "wireless network*" or radiowave* or radio-wave* or microwave* or micro-wave* or powerline* or gsm or umts or lte or 5g or mhz or ghz or "millimeter wave*" or "millimetre wave*" or "mm wave*")

18 2,805

TS=(tablet* NEAR/3 (device* or computer*))

17 4,661

TS=(ipad* or i-pad* or ipod* or i-pod* or iphone* or i-phone*)

16 276,026

TS=(smartphone* or smart-phone* or cellphone* or mobiles)

15 63,111

TS=((cell* or mobile* or cordless) NEAR/1 (phone* or telephon* or technolog* or device*))

14 5,518,820

#13 OR #12 OR #11 OR #10

13 902,981

TS=((before NEAR/5 after) or (pre NEAR/5 post) or pretest or "pre test" or posttest or "post test" or quasiexperiment* or "quasi experiment*" or double-blind* or sham or ((cross-over or crossover) NEAR/2 (study or design or trial)))

12 4,746,478

AB=(trial or groups)

11 366,838

AB=randomly

10 555,083

AB=randomi?ed

9 2,979,677

#8 OR #7 OR #6 OR #5 OR #4 OR #3 OR #2 OR #1

8 813,974

TS=(risk* NEAR/2 factor*)

7 67

TS="case reference"

6 91,575

TS=((Ecologic* or Panel) NEAR (study or studies))

5 269,980

TS=("time series" or "time point*")

4 1,144,464

TS=(Retrospective or prospective or sham)

3 674,625

TS=((observational or longitudinal or field) NEAR (study or studies))

2 154,648

TS=("Follow up" NEAR (study or studies))

1 479,112

TS=((cohort or case-control) NEAR (study or studies or analy*))

2. **Ovid MEDLINE(R) and Epub Ahead of Print, In-Process & Other Non-Indexed Citations, Daily and Versions(R) <1946 to February 03, 2021>Searched 4th February 2021**

1 Epidemiologic studies/ (8561)
2 exp case control studies/ (1142070)
3 exp cohort studies/ (2089253)
4 ((cohort or case-control) adj (study or studies or analy*)).tw. (340827)
5 Follow-up Studies/ (656174)
6 (Follow up adj (study or studies)).tw. (50643)
7 observational study/ (92854)
8 ((observational or field) adj (study or studies)).tw. (133338)
9 Longitudinal studies/ (141898)
10 Longitudinal.tw. (260874)
11 Retrospective studies/ or Prospective studies/ (1409797)
12 (Retrospective or prospective or sham).tw. (1218146)
13 Interrupted Time Series Analysis/ (1126)

14 (time series or time point?).ti,ab. (155203)
15 ((Ecologic* or Panel) adj (study or studies)).tw. (8666)
16 "case reference".tw. (57)
17 risk factors/ or (factors adj2 risk*).tw. (1077420)
18 or/1-17 (3817334)
19 randomized controlled trial/ or equivalence trial/ or pragmatic clinical trial/ (523828)
20 controlled clinical trial/ or double-blind method/ or cross-over studies/ (276303)
21 (randomi?ed or randomly or trial or groups).ab. (2946745)
22 drug therapy.fs. (2278193)
23 ((before adj5 after) or (pre adj5 post) or pretest or pre test or posttest or post test or quasiexperiment* or quasi experiment* or double-blind* or sham or ((cross-over or crossover) adj2 (study or design or trial))).ti,ab. (764581)
24 or/19-23 (5290716)
25 telephone/ or exp cell phone/ (23179)
26 "Cell Phone Use"/ae, sn, td [Adverse Effects, Statistics & Numerical Data, Trends] (127)
27 computers, handheld/ or smartphone/ (8875)
28 ((cell* or mobile* or cordless) adj1 (phone* or telephon* or technolog* or device*)).ti,ab,kw. (20901)
29 (smartphone* or smart-phone* or cellphone* or mobiles).ti,ab,kw. (15344)
30 (ipad* or i-pad* or ipod* or i-pod* or iphone* or i-phone*).ti,ab,kw. (3043)
31 (tablet* adj3 (device* or computer*)).ti,ab,kw. (1808)
32 Magnetic Fields/ae or Electromagnetic Phenomena/ae, sn or Electromagnetic Fields/ae or Electromagnetic Radiation/ or Radio Waves/ae or Microwaves/ae, sn or Wireless Technology/lj, st, sn, td (7505)
33 (electromagnetic or electro-magnetic or radiofrequency or radio-frequency or DECT or "cordless telecomm*" or ((radio or telephon* or mobile or base or radar or broadcast* or television or tv) adj2 (station* or mast or masts or transmitter* or antenna*)) or TETRA or "plastic sealer*" or wifi or wi-fi or wlan or "wireless area network*" or "wireless network*" or radiowave* or radio-wave* or microwave* or micro-wave* or powerline* or gsm or umts or lte or 5g or mhz or ghz or "millimeter wave*" or "millimetre wave*" or "mm wave*").ti,ab,kw. (175892)
34 or/25-33 (230491)
35 Cognition/re [Radiation Effects] (473)
36 cognition disorders/ep, et, pp or auditory perceptual disorders/ep, et, pp (39376)
37 Sleep/re [Radiation Effects] (195)
38 Sleep Deprivation/ep, et, pp or Sleep Wake Disorders/ep, et, pp or Sensory Deprivation/et, ph, pp (19087)
39 memory disorders/ep, et, pp or perceptual disorders/ep, et, pp or sensation disorders/ep, et, pp or hearing disorders/ep, et, pp or somatosensory disorders/ep, et, pp or dizziness/ep, et, pp (30342)
40 Auditory Perception/ or Perception/ (64347)
41 Self Report/ (35006)
42 Fatigue/ep, et, pp or Mental Fatigue/ep, et, pp (16102)
43 Tinnitus/ep, et, pp (4659)
44 headache/ep, et, pp or psychophysiologic disorders/ep, et, pp (20486)
45 nocebo effect/ (304)
46 Hypersensitivity/ep, et, pp (11662)
47 exp Hearing Loss/ep, et, pp (29604)
48 Affective Symptoms/ep, et, pp or neurasthenia/ep, et, pp (4736)
49 (electrohypersensitiv* or IEI-EMF or "environmental intolerance" or electrosensitiv* or "electric* sensitivity" or sleep* or memory or hearing or auditory or percept* or perceiv* or self-report* or "self report*" or dizziness or dizzy or fatigue or tiredness or tinnitus or headache* or nocebo or psychophysiologic* or psychosomatic or psycho-somatic or hypersensitiv* or well-being or "well being" or "quality of life" or HRqol or (concentrat* adj2 (abilit* or inability or capacit*)) or nervous or neurasthen* or vegetative or ((body or bodily or physical or physiological* or unspecifi* or non-specifi* or indetermin* or undetermin*) adj3 (sensation* or feeling* or symptom* or change*))).ti,kw. (703090)

50 or/35-49 (854869)
51 exp animals/ not humans.sh. (4786715)
52 18 and 34 and 50 (1525)
53 52 not 51 (1463)
54 24 and 34 and 50 (2139)
55 54 not 51 (2018)
56 53 or 55 (2794)
57 (((messag* or text or texts or texting or sms or app) adj5 (treat* or therap* or cessation)) or mhealth or m-health or "mobile health" or ablation* or transcranial* or trans-cranial* or (adher* adj3 (therap* or treatment))).ti,ab,kw. (168602)
58 Text Messaging/ (3221)
59 exp Telemedicine/ (32585)
60 exp Catheter Ablation/ or exp Ablation Techniques/ or exp Radiofrequency Ablation/ or exp High-Intensity Focused Ultrasound Ablation/ (117756)
61 magnetic field therapy/ or transcranial magnetic stimulation/ (13071)
62 Transcranial Direct Current Stimulation/ or Ultrasonography, Doppler, Transcranial/ (10508)
63 or/57-62 (284497)
64 56 not 63 (**2052**)
