# Supplementary material for: The effects of radiofrequency electromagnetic fields exposure on tinnitus, migraine and non-specific symptoms in the general and working population: A protocol for a systematic review on human observational studies
Source: Environ Int. 2021 Dec;157:106852. doi: 10.1016/j.envint.2021.106852 (PMC8484767; doi:10.1016/j.envint.2021.106852)
Supplement: Supplementary data 3 [file mmc3.pdf]

Appendix C: Risk of Bias Tool for a systematic review of the effects of radiofrequency electromagnetic fields exposure on symptoms: human observational research

For the Risk of Bias assessment, we have adapted the OHAT Risk of Bias Rating Tool for Human and Animal Studies (NTP 2015) to the specific circumstances of observational studies on electromagnetic field exposure and symptom development. As indicated in the OHAT tool we customized the Risk of Bias tool during the protocol development. We only kept the questions related to cohort (**Co**) and case-control studies (**CaCo**) as suggested by OHAT. Further, we took special attention to tailor the Risk of Bias tool for the following three key criteria for 3-Tier system: 1) consideration of potential confounders, 2) confidence in the exposure characterization, and 3) confidence in the outcome assessment.
In this document we have copied the original OHAT Risk of bias tool (black font). The reviewer is expected to apply the OHAT Risk of bias tool in the sense of the original meaning. However, sometimes the wording is not specific for the RF-EMF research area or specific circumstances are not described. Thus, any further explanations and specifications made to the instruction section in addition to the original OHAT tool are highlighted in blue and should be considered in the risk of bias analysis.

Format of the Rating instructions:

- Each question of the background section contains the following information
    - Definition of the general category of bias
    - Clarifying text to explain what study aspects are relevant
    - Available empirical information about the direction and magnitude of the bias
    - Information about other internal validity assessment tools that consider this element
- Specific risk-of-bias rating instructions customized to each study type
    - Detailed criteria are outlined that define aspects of the study design, conduct, and reporting required to reach each risk-of-bias rating
    - The criteria are focused on distinguishing among the 4 risk-of-bias answers or ratings (e.g., outlining factors that separate "definitely low" from "probably low" risk of bias
- Further explanations and instructions targeted to our review added by the study team
    - Specific criteria relevant for the available literature

Answer Format:

++ ***Definitely Low*** *risk of bias*:

There is **direct evidence** of low risk-of-bias practices
(May include specific examples of relevant low risk-of-bias practices)

\+ ***Probably Low*** *risk of bias*:

There is **indirect evidence** of low risk-of-bias practices **OR** it is deemed that **deviations from low risk-of-bias practices for these criteria during the study would not appreciably bias results, including consideration of direction and magnitude of bias**.

– NR ***Probably High*** *risk of bias*:

There is **indirect evidence** of high risk-of-bias practices **OR** there is **insufficient information (e.g., not reported or "NR")** provided about relevant risk-of-bias practices

–– ***Definitely High*** *risk of bias*:

There is **direct evidence** of high risk-of-bias practices
(May include specific examples of relevant high risk-of-bias practices)

The system for answering each risk-of-bias question requires reviewers to choose between low and high risk-of-bias options. This 4-point scale is based on the approach taken by the Clarity Group at McMaster University without an answer for mixed or unclear evidence (2013). A conservative approach is taken wherein insufficient information to clearly judge the risk of bias for an individual question results in an answer rating of "Probably High" risk of bias. To clearly identify answers that were reached due to insufficient information, there are two separate symbols for "Probably High" risk of bias: 1) "-" for indirect evidence of high risk-of-bias practices, and 2) "NR" (=not reported) when there is insufficient information. The general answer format was adapted from (Koustas *et al.* 2013).

Direction of Bias

Empirical evidence about the direction of bias is discussed for each of the risk-of-bias questions. For some questions, the evidence will be easier to evaluate as toward or away from the null. For example, non-differential unintended co-exposure to high background phytoestrogen content in the diet will bias experimental studies of low-dose estrogenic effects toward the null. However, if there is no clear rationale for judging the likely direction of bias, review authors should simply outline the evidence and not attempt to guess the direction of evidence (Sterne *et al.* 2014).

For each bias assessment that resulted in probably or definitively high risk of bias, the reviewer is required do an overall judgement for the direction of the bias for the corresponding effect estimate. This includes the following four answer formats:

- false positive risk (overestimation)
- false protective finding (overestimation benefits)
- bias towards absence of an association
- unpredictable.

Overview of selected risk of bias questions

(A) Selection Bias

1. Did **selection** of study participants result in appropriate comparison groups?

(B) Confounding Bias

2. Did the study design or analysis account for important **confounding** and modifying variables?

(C) Attrition/Exclusion Bias (ALL)

3. Were outcome data complete without **attrition or exclusion** from analysis?

(D) Detection Bias (ALL)

4. Can we be confident in the **exposure characterization**?
5. Can we be confident in the **outcome assessment**?

(E) Selective Reporting Bias (ALL)

6. Were all measured **outcomes** reported?

(F) Other Bias

7. Were there no other potential threats to **internal validity** (e.g., statistical methods were appropriate and researchers adhered to the study protocol)?

   7a. Were statistical methods appropriate?

   7b. Is there evidence for reverse causality?

RISK OF BIAS RATING INSTRUCTIONS

(A) Selection Bias

Selection bias refers to systematic differences between baseline characteristics of the groups that are compared (Higgins and Green 2011).

1. Did selection of study participants result in appropriate comparison groups?

Comparison group appropriateness refers to having similar baseline characteristics of factors related to the outcome measures of interest between groups aside from the exposures (and outcomes for case-control studies).

Assessment of appropriate selection of comparison groups is a widely used element of tools to assess study quality for observational human studies (Downs and Black 1998, Shamliyan *et al.* 2010, Viswanathan *et al.* 2012, CLARITY Group at McMaster University 2013, Sterne *et al.* 2014, Wells *et al.* 2014). This question addresses whether exposed and unexposed subjects were recruited from the same populations in cohort or cross-sectional studies and consideration of appropriate selection of cases and controls in case-control studies.

The direction of the bias (towards or away from the null) will differ based on the nature of differences between comparison groups and may be difficult to predict.
For example, in occupational cohorts, it is common for workers to have lower rates of disease and mortality than the general population – the healthy worker effect – because the severely ill and chronically disabled are commonly excluded from employment (Gerstman 2013). Therefore, comparing workers to an inherently less healthy group (general population or workers with less physically demanding work) can bias the estimate of disease risk towards the null (Rothman *et al.* 2012). Conversely, if cases of disease identified from a screening program were compared to controls from the general population, the effect estimate could be overestimated as those being screened may inherently have a higher risk (e.g., family history) so the better comparison group would be subjects screened as not having disease (Szklo and Nieto 2007).
For controlled exposure studies (i.e., experimental human or animal studies), the potential for imbalance of baseline characteristics is controlled for through randomization and allocation concealment. Imbalance can arise from chance alone, but baseline characteristics should be similar for truly randomized human controlled trials (Higgins and Green 2011) or other experimental studies. The majority of study quality tools for experimental animals do not have a separate question on baseline characteristics (Krauth *et al.* 2013, Koustas *et al.* 2014); although the SYRCLE tool asks whether groups were "similar at baseline or were they adjusted for confounders in the analysis" (Hooijmans *et al.* 2014). The Cochrane risk-of-bias tool for randomized controlled trials does not include a routine question on baseline characteristics, and instead suggests that reviewers consider "inexplicable baseline imbalance" under other potential threats to internal validity (Higgins *et al.* 2011). This tool takes the same approach for all experimental studies and addresses baseline imbalance for these studies only where it is strongly suspected with a question at the end of the risk-of-bias-tool under other potential threats to internal validity.

In principle, the most critical issue to consider is that the likelihood to participate in the study did not depend on both: exposure status and risk profile for the outcome of interest. Unfortunately, the exposure status of non-participating individuals is usually not known. In cohort studies, participation rate at baseline is less critical for the internal validity of the study. As long as inclusion and exclusion criteria, recruitment and ascertainment methods do not systematically differ between health and exposure status, even a low participation rate is considered to be of probably low risk of bias. However, probably or definitively high risk of bias may occur in cohort studies recruiting from very narrow exposure settings, like comparing individuals from exposed community A to non-exposed community B, independent of the participation rate, in particular if there is evidence that the study was set up as a response to complaints.
For case-control studies, it is required that cases and controls were recruited from the same eligible population, including being of similar age, gender, ethnicity. It is also important that cases and controls are included in the study within the same time frame. Difference in participation rates between cases and controls makes the study more vulnerable to selection bias.

Definitely Low risk of bias:

Co: There is direct evidence that subjects (irrespective of exposure status) were similar (e.g., recruited from the same eligible population, recruited with the same method of ascertainment using the same inclusion and exclusion criteria, and were of similar age and health status), recruited within the same time frame, and had similar participation/response rates.

CaCo: There is direct evidence that cases and controls were similar (e.g., recruited from the same eligible population including being of similar age, gender, ethnicity, and eligibility criteria other than outcome of interest as appropriate), recruited within the same time frame, and

controls are described as having no history of the outcome

AND probability of participation is unlikely to be associated with the exposure and the case/control status.

Note: A study will be considered low risk of bias if baseline characteristics of groups differed but these differences were considered as potential confounding or stratification variables (see question #2).

Probably Low risk of bias:

Co: There is indirect evidence that subjects (irrespective of exposure status) were similar (e.g., recruited from the same eligible population, recruited with the same method of ascertainment using the same inclusion and exclusion criteria, and were of similar age and health status), recruited within the same time frame, and had similar participation/response rates,

OR differences between groups would not appreciably bias results.

CaCo: There is indirect evidence that cases and controls were similar (e.g., recruited from the same eligible population, recruited with the same method of ascertainment using the same inclusion and exclusion criteria, and were of similar age), recruited within the same time frame, and controls are described as having no history of the outcome,

AND probability of participation is unlikely to be associated with the exposure and the case/control status.

Probably High risk of bias:

Co: There is indirect evidence that subjects (irrespective of exposure status) were not similar, recruited within very different time frames, or had different participation/response rates,

OR there is insufficient information provided about the comparison group including a different rate of non-response without an explanation (record "NR" as basis for answer).

CaCo: There is direct evidence that controls were drawn from a very dissimilar population than cases or recruited within very different time frames,

OR probability of participation likely to be associated with the exposure,

OR there is insufficient information provided about the appropriateness of controls including rate of response reported for cases only (record "NR" as basis for answer).

Definitely High risk of bias:

Co: There is direct evidence that subjects (both exposed and non-exposed) were not similar, recruited within very different time frames, or had very different participation/response rates.

CaCo: There is direct evidence that controls were drawn from a very dissimilar population than cases or recruited within very different time frames.

OR that probability of participation depended on both, the exposure and the case/control status.

(B) Confounding Bias

Bias relating to confounding and co-exposures is addressed under selection bias and performance in study quality tools such as Cochrane, AHRQ, and SYRCLE (Higgins and Green 2011, Higgins *et al.* 2011, Viswanathan *et al.* 2012, Hooijmans *et al.* 2014). The grouping of these related factors under "confounding bias" does not change the questions or the evaluation of bias, but rather is done for clarity in communicating bias related to confounding, modifying variables, and other exposures that are anticipated to bias results.

2. Did the study design or analysis account for important confounding and modifying variables?

Interpretation of study findings may be distorted by failure to consider the extent to which systematic differences in baseline characteristics risk factors, prognostic variables, or co-occurring exposures among comparison groups may reduce or increase the observed effect (IOM 2011). Confounding variables or confounders include any factor that is: 1) associated with the exposure, 2) an independent risk factor for a given outcome, and 3) unequally distributed between study groups (Gerstman 2013). The potential confounder cannot be an intermediate effect on the causal pathway between exposure and the outcome (Gerstman 2013, Sterne *et al.* 2014). Appropriate methods to account for these differences would include multivariable analysis, stratification, matching of cases and controls, or other approaches.

Adjusting or controlling for confounding is dependent on valid, reliable, and sensitive methods for assessing the confounding or modifying variables applied consistently across study groups. The requirement for assessing the confounding variables with valid and reliable measures is directly linked to the relative importance of the confounding variable considered under selection bias (i.e., if a confounder needed to be accounted for in design or analyses, then measurement of that variable had to be reliable).

This element is included in this current risk-of-bias tool because it is widely recommended in tools used to assess the quality of observational human studies (Downs and Black 1998, Shamliyan *et al.* 2010, Viswanathan *et al.* 2012, CLARITY Group at McMaster University 2013, Viswanathan *et al.* 2013, Sterne *et al.* 2014). The direction of the bias (towards or away from the null) will differ based on the nature of differences between comparison groups. Generally, confounding results in effect sizes that are overestimated. However, confounding factors can lead to an underestimation of the effect of a treatment or exposure, particularly in observational studies. In other words, if the confounding variables were not present, the measured effect would have been even larger (IOM 2011).

Unintended co-exposures may represent a confounding factor if associated with exposure and the outcome of interest, or a modifying factor if they are independent of exposure, but associated with outcome. When an unintended exposure is an effect modifier, its level will alter the magnitude of the effect of the primary outcome. The direction of the bias (towards or away from the null) will differ based on the nature of unintended exposure and whether or not it is associated with the primary exposure. For example, an exposed group in a human study living at a Superfund site may also be exposed to high levels of other environmental contaminants; if these co-exposures are not accounted for in the analyses, they may bias results away from the null (towards larger effects sizes). Alternately, a co-exposure that is non-differentially distributed among both the exposed and control groups will usually bias the results toward the null by lowering precision and therefore reducing the ability to distinguish potential effects between groups based on the primary exposure.

It is understood in environmental health that people are exposed to complex mixtures of environmental contaminants and other types of exposures that make it difficult to establish chemical-specific associations. Thus, in most cases we will not penalize studies if other exposures or potential exposures are not adjusted or controlled for in the analyses of a target exposure. For some projects, exceptions may include studies where levels of

other chemicals aside from the chemical of interest are likely to be high, such as in occupational cohorts or contaminated regions (e.g., Superfund sites). For some health outcomes, consideration of additional therapies, including medications, may also be appropriate.

By definition, confounders are specific for the outcome and the exposure. Therefore, the list of potential confounders has to be developed specifically for each evaluation and will require subject-matter expertise on both the outcome and exposure of interest. Systematic review authorities recommended that subject-matter experts with some knowledge of the literature participate in drafting a list of potential confounders when a review protocol is developed (Viswanathan *et al.* 2013, Sterne *et al.* 2014). It may be helpful to draft an analytic framework that shows potential confounders that could affect the relationship between exposure and outcomes of interest. Even when a list of potential confounders is developed when drafting the protocol, it is likely that new confounders will be identified when actually assessing the risk of bias of studies.

Although confounding is a much greater concern for observational studies, experimental studies are not entirely free of these issues. Controlled exposure studies (i.e., experimental human or animal studies) can address confounding and selection bias through study design features such as randomization and allocation concealment. Confounding by chance (i.e., confounding that is unknown, unmeasured, or poorly measured) is expected to be equally distributed between groups under true randomization; however, experimental studies may not always successfully randomize potential confounders (Viswanathan *et al.* 2013). Recognizing this, the SYRCLE risk-of-bias tool for experimental animal studies asks whether groups were "similar at baseline or were they adjusted for confounders in the analysis" (Hooijmans *et al.* 2014). The 2012 risk-of-bias guidance from AHRQ recommends consideration of confounding for randomized clinical trials largely because studies may fail to randomize confounders. However, the Cochrane risk-of-bias tool for randomized controlled trials does not include a question for confounding, nor do the majority of study quality tools for experimental animals (Krauth *et al.* 2013, Koustas *et al.* 2014).

For this tool, we have not included a separate question for confounding in experimental human or experimental animal studies because randomization and allocation concealment should address the issue of confounding. Therefore, the issue of confounding overlaps with randomization and allocation concealment, and multiple questions would address the same issue. We recognize that in some cases confounding or effect modification may be a potential risk of bias despite procedures to address randomization. For example, confounding would be a concern if there were differential distribution of baseline characteristics such as body weight or BMI in a study of obesity, despite adequate procedures for randomization and allocation concealment. In another example, effect modification and bias toward the null would be of concern in an experimental study designed to test reproductive effects of estrogenic chemicals with non-differential co-exposures to high levels of phytoestrogens through the diet. For experimental studies where confounding is strongly suspected, randomization and allocation concealment should be addressed first. If these questions are rated "probably low" or "definitely low risk of bias," then confounding may be addressed under "other potential threats to internal validity."

Note: in the current OHAT tool, assessment of confounding requires consideration of whether or not 1) the design or analysis accounted for confounding and modifying variables, 2) the confounding variables were measured reliably and consistently, and 3) there were other exposures anticipated to bias results in reaching a single risk-of-bias rating on confounding. Previous versions of the OHAT tool used three separate questions for these factors (Did the study design or analysis account for important confounding and modifying variables?" "Were confounding variables assessed consistently across groups using valid and reliable measures" and "Did researchers adjust or control for other exposures that are anticipated to bias results?" The current tool considers these factors together because they are interrelated and recent guidance has taken a similar approach (e.g., Sterne et al. 2014).

Observational studies on near field exposure are vulnerable to numerous confounding factors because many risk and protective factors for symptoms may also be related to the use of such e-media devices and thus correlated with RF-EMF exposure. Further, it is conceivable that e-media use is related to latent variables, which act as confounders by indication or are causing reverse causality that may even be relevant for longitudinal studies but this will be considered in item 7b. It is considered impossible to accurately identify an exhaustive list of all conceivable relevant confounding factors for various outcomes, because this depends on the underlying biological mechanism, which is unknown. Nevertheless, **the following confounders are minimally required: sex, age, a measure of socioeconomic status and a measure of distress.** Further, the strategy to minimize confounding should be evaluated in terms of general design of the study, selected covariates matching, stratification, propensity scoring or other methods and sensitivity that are appropriately justified to evaluate the robustness of the associations. By definition, observational studies on near field exposure should not receive a “definitive low risk of bias” evaluation.
Studies related to far-field exposure are less vulnerable to confounding from lifestyle factors. **Thus, the minimal set of confounders includes sex, age, and a measure of socioeconomic status.** However, these studies need to consider the plausibility that the exposure of interest is relevant for the total RF-EMF exposure at all. If it is only a small proportion, the likelihood of bias is increased and should not receive a “definitive low risk of bias”. If far field exposure is masked by substantially higher exposure from near field exposure, and this is not considered in the data analysis, a bias towards absence of association may occur. Further, any study that compares two differently exposed areas is prone to probable or definitive risk of bias from confounding.
For occupational exposure studies the following minimal confounders needs to be considered: sex, age, a measure of socioeconomic status and a measure of distress. Comparison of two differently exposed exposure group results in probable or definitive high risk of bias from confounding by design.

Definitely Low risk of bias:

Co: There is direct evidence that appropriate adjustments or explicit considerations were made for primary covariates and confounders in the final analyses through the use of statistical models to reduce research-specific bias including standardization, matching, adjustment in multivariate model, stratification, propensity scoring, or other methods that were appropriately justified. Acceptable consideration of appropriate adjustment factors includes cases when the factor is not included in the final adjustment model because the author conducted analyses that indicated it did not need to be included,

AND there is direct evidence that primary covariates and confounders were assessed using valid and reliable measurements,

AND there is direct evidence that other exposures anticipated to bias results were not present or were appropriately measured and adjusted for. In occupational studies or studies of contaminated sites, other chemical exposures known to be associated with those settings were appropriately considered.

CaCo: There is direct evidence that appropriate adjustments were made for primary covariates and confounders in the final analyses through the use of statistical models to reduce research-specific bias including standardization, matching of cases and controls, adjustment in multivariate model, stratification, propensity scoring, or other methods were appropriately justified,

AND there is direct evidence that primary covariates and confounders were assessed using valid and reliable measurements,

AND there is direct evidence that other exposures anticipated to bias results were not present or were appropriately measured and adjusted for.

Note: No observational study in this field of research has the potential for definitive low risk of bias from confounding.

Probably Low risk of bias:

Co, CaCo: There is indirect evidence that appropriate adjustments were made,

OR it is deemed that not considering or only considering a partial list of covariates or confounders in the final analyses would not appreciably bias results.

AND there is evidence (direct or indirect) that primary covariates and confounders were assessed using valid and reliable measurements**,**

OR it is deemed that the measures used would not appreciably bias results (i.e., the authors justified the validity of the measures from previously published research),

AND there is evidence (direct or indirect) that other co-exposures anticipated to bias results were not present or were appropriately adjusted for,

OR it is deemed that co-exposures present would not appreciably bias results.

Note: As discussed above, this includes insufficient information provided on co-exposures in general population studies.

Note: Convincing methods and convincing data are presented to evaluate the relevance of confounding which includes (but is not restricted to) results of sensitivity analysis for different covariable selection and comparison of basic and fully adjusted analysis models.

Near field and **occupational** exposure studies should have used appropriate methods to check that exposure of interest does not affect the outcome by a non-RF-EMF related mechanism. In addition to confounding adjustment this should be done by calculating dose or use of negative exposure control variable.

Probably High risk of bias:

Co: There is indirect evidence that the distribution of primary covariates and known confounders differed between the groups and was not appropriately adjusted for in the final analyses,

OR there is insufficient information provided about the distribution of known confounders (record "NR" as basis for answer),

OR there is indirect evidence that primary covariates and confounders were assessed using measurements of unknown validity,

OR there is insufficient information provided about the measurement techniques used to assess primary covariates and confounders (record "NR" as basis for answer),

OR there is indirect evidence that there was an unbalanced provision of additional co-exposures across the primary study groups, which were not appropriately adjusted for,

OR there is insufficient information provided about co-exposures in occupational studies or studies of contaminated sites where high exposures to other chemical exposures would have been reasonably anticipated (record "NR" as basis for answer).

CaCo: There is indirect evidence that the distribution of primary covariates and known confounders differed between cases and controls and was not investigated further,

OR there is insufficient information provided about the distribution of known confounders in cases and controls (record "NR" as basis for answer),

OR there is indirect evidence that primary covariates and confounders were assessed using measurements of unknown validity,

OR there is insufficient information provided about the measurement techniques used (record "NR" as basis for answer),

OR there is indirect evidence that there was an unbalanced provision of additional co-exposures across cases and controls, which were not appropriately adjusted for,

OR there is insufficient information provided about co-exposures in occupational studies or studies of contaminated sites where high exposures to other chemical exposures would have been reasonably anticipated (record "NR" as basis for answer).

Note: Studies that did not include the minimal set of confounders as defined above are considered to be of probable or definitive high risk of bias.

Definitely High risk of bias:

Co: There is direct evidence that the distribution of primary covariates and known confounders differed between the groups, confounding was demonstrated, and was not appropriately adjusted for in the final analyses,

OR there is direct evidence that primary covariates and confounders were assessed using non valid measurements,

OR there is direct evidence that there was an unbalanced provision of additional co-exposures across the primary study groups, which were not appropriately adjusted for.

CaCo: There is direct evidence that the distribution of primary covariates and known confounders differed between cases and controls, confounding was demonstrated, but was not appropriately adjusted for in the final analyses,

OR there is direct evidence that primary covariates and confounders were assessed using non valid measurements,

OR there is direct evidence that there was an unbalanced provision of additional co-exposures across cases and controls, which were not appropriately adjusted for.

(C) Attrition/Exclusion Bias (ALL)

Attrition or exclusion bias refers to systematic differences in the loss or exclusion from analyses of participants or animals from the study and how they were accounted for in the results (Viswanathan *et al.* 2012).

3. Were outcome data complete without attrition or exclusion from analysis?

Incomplete outcome data includes loss due to attrition (nonresponse, dropout, or loss to follow-up) or exclusion from analyses. The degree of bias resulting from incomplete outcome data depends on the reasons that outcomes are missing, the amount and distribution of missing data across groups, and the potential association between outcome values and likelihood of missing data (Higgins and Green 2011). The risk of bias from incomplete outcome data can be reduced if study authors address the problem in their analyses (e.g., intention to treat analysis and imputation). Exclusion of individuals or animals from analyses should be clearly reported and outliers identified with appropriate statistical procedures.

Human introductory text: Differential or overall attrition because of nonresponse, dropping out, loss to follow-up, and exclusion of participants can introduce bias when missing outcome data are related to both exposure/treatment and outcome. Those who drop out of the study or who are lost

to follow-up may be systematically different from those who remain in the study. Attrition or exclusion bias can potentially change the collective (group) characteristics of the relevant groups and their observed outcomes in ways that affect study results by confounding and spurious associations (Viswanathan *et al.* 2012). This risk-of-bias element is recommended to assess controlled human trials (Higgins and Green 2011), observational human studies (Viswanathan *et al.* 2012, Sterne *et al.* 2014) and animal studies (Krauth *et al.* 2013). However, concern over bias from incomplete outcome data is mainly theoretical and most studies that have looked at whether aspects of missing data are associated with magnitude of effect estimates have not found clear evidence of bias (reviewed in Higgins and Green 2011).

In this field of research, a concern is that individuals reporting to react strongly to RF-EMF exposure (e.g. EHS individuals) may show a different attrition than the general population. It should thus be specifically evaluated whether it has been reported or seems plausible that EHS individuals have a different chance to stay in the study or to drop out. Another aspect to consider in cohort and case-control studies is whether missing information in confounding variables has resulted in dropouts from the original study population. Multiple imputations and similar techniques to deal with missing observations in some variables are considered to result in a probably low risk of bias.

Definitely Low risk of bias:

Co: There is direct evidence that loss of subjects (i.e., incomplete outcome data) was adequately addressed and reasons were documented when human subjects were removed from a study. Acceptable handling of subject attrition includes: very little missing outcome data (ca. ≤20%); reasons for missing subjects unlikely to be related to outcome (for survival data, censoring unlikely to be introducing bias); missing outcome data balanced in numbers across study groups, with similar reasons for missing data across groups,

OR missing data have been imputed using appropriate methods and characteristics of subjects lost to follow up or with unavailable records are described in identical way and are not significantly different from those of the study participants.

CaCo: There is direct evidence that exclusion of subjects from analyses was adequately addressed, and reasons were documented when subjects were removed from the study or excluded from analyses.

Probably Low risk of bias:

Co: There is indirect evidence that loss of subjects (i.e., incomplete outcome data) was adequately addressed and reasons were documented when human subjects were removed from a study,

OR it is deemed that the proportion lost to follow-up would not appreciably bias results. This would include reports of no statistical differences in characteristics of subjects lost to follow up or with unavailable records from those of the study participants. Generally, the higher the ratio of participants with missing data to participants with events, the greater potential there is for bias. For studies with a long duration of follow-up, some withdrawals for such reasons are inevitable.

CaCo: There is indirect evidence that exclusion of subjects from analyses was adequately addressed, and reasons were documented when subjects were removed from the study or excluded from analyses.

Probably High risk of bias:

Co: There is indirect evidence that loss of subjects (i.e., incomplete outcome data) was unacceptably large (in the range of >20%) and not

adequately addressed,

OR there is insufficient information provided about numbers of subjects lost to follow-up (record "NR" as basis for answer).

CaCo: There is indirect evidence that exclusion of subjects from analyses was not adequately addressed,

OR there is insufficient information provided about why subjects were removed from the study or excluded from analyses (record "NR" as basis for answer).

Definitely High risk of bias:

Co: There is direct evidence that loss of subjects (i.e., incomplete outcome data) was unacceptably large (ca. >20%) and not adequately addressed. Unacceptable handling of subject attrition includes: reason for missing outcome data likely to be related to true outcome, with either imbalance in numbers or reasons for missing data across study groups; or potentially inappropriate application of imputation.

CaCo: There is direct evidence that exclusion of subjects from analyses was not adequately addressed. Unacceptable handling of subject exclusion from analyses includes: reason for exclusion likely to be related to true outcome, with either imbalance in numbers or reasons for exclusion across study groups.

(D)Detection Bias (ALL)

Detection bias refers to systematic differences between experimental and control groups with regards to how outcomes and exposures are assessed (Higgins and Green 2011) and also considers validity and reliability of methods used to assess outcomes and exposures (Viswanathan *et al.* 2012).

4. Can we be confident in the exposure characterization?

Confidence in the exposure requires valid, reliable, and sensitive methods to measure exposure applied consistently across groups. Exposure misclassification or measurement error may be independent of the outcomes (non-differential) or related to the outcome of interest (differential). Non-differential measurement error of exposures will usually bias the results toward the null by lowering precision and therefore reducing the ability to distinguish potential effects between exposure levels. Therefore, this tool considers the accuracy of the exposure characterization, including both purity and stability for controlled exposure studies, as part of the risk-of-bias rating for exposure. Differential measurement error of exposures can bias the exposure-outcome relationship and result in detection bias.

Detection bias can be minimized by using valid and reliable exposure measures applied consistently across groups (i.e., under the same method and time-frame). Studies that directly measure exposure in subjects (e.g., measurement of the chemical in blood, plasma, urine, etc.) are likely to have less measurement error and less risk of bias for exposure than studies relying on indirect measures (e.g., predictions from activity patterns and microenvironment concentrations). Exposure information obtained by self-report depends on the recall of participants and differential errors in recall can attenuate, strengthen, or even invert the true relationship (White 2003). Self-reporting of exposures for case-control studies are frequently cited as leading to differential measurement errors because cases often remember past exposures better than controls (i.e., recall bias) (e.g., see Rothman *et al.* 2012). Differential measurement error could also be introduced if the exposure data for different groups come from different sources for

observational studies or are taken at different time points for experimental studies.

Acceptable methods for measuring exposure will be highly exposure dependent and therefore a specific list of acceptable, inaccurate, or potentially biased methods should be developed for each evaluation and will require subject-matter expertise. It is recommended that experts with some knowledge of the literature (including exposure and outcomes) participate in drafting the risk-of-bias criteria for exposure characterization when a review protocol is developed. Even with early expert consultation and planning, exposure questions may arise when the actual studies are assessed. Additional consultation and modifications to the exposure risk-of-bias criteria may be necessary. When changes are made, they should be documented along with the date on which modifications were made and the logic for the changes.

For controlled exposure studies (i.e., experimental human or animal studies), the use of reliable methods to measure exposure depends primarily on ensuring the purity and stability of the treatment compound. Independent verification of purity would be considered best practice because the identity and purity as listed on the bottle can be inaccurate. In NTP's experience, about 3% of chemicals purchased are the wrong chemical and the inaccuracy rate of chemical labelling rises to 10% if you include inaccurate reporting of purity (unpublished, personal communication Brad Collins, NTP chemist). It is also possible that impurities may be more toxic than the compound of interest. This occurred during an NTP study of PCB 118 where analysis revealed the presence of 0.622% of the much more potent PCB 126, resulting in the study being continued as a mixture study [(NTP 2006), see page 13]. The directions below takes a conservative approach in requiring independent verification of ≥99% purity for a single substance for "definitely low" risk of bias. However, the risk of bias associated with exposure to impurities depends on the identity of the impurities and the sensitivity of the outcome of interest which could result in potential effects of those impurities on the outcome of interest. The threshold for these values should be developed for specific research questions and reflect empirical data for the substance and outcome under consideration when possible. Therefore, for some chemicals like PCBs, ≥99% purity may not be sufficient for "definitely low" risk of bias and for others the appropriate purity value may be lower.

Exposure characterization should also include verification of the compound over the course of the test period. This is particularly important if the compound is volatile or instable. For example, daily preparation of treatment solutions may be required for unstable compounds (e.g., half-lives on the order of days). Special apparatus such as flow-through systems are needed to ensure exposure to volatile compounds. For example, Durda and Preziosi (2000) suggest the use of flow-through systems in aquatic exposures to volatile compounds (e.g., those with Henry's Law values in the range of 10^{-5} atm- m^3/mol or greater).

Human introductory text: Assessment of exposure is a widely used element of tools to assess study quality for observational human studies (Downs and Black 1998, Shamliyan *et al.* 2010, Viswanathan *et al.* 2012, CLARITY Group at McMaster University 2013, Wells *et al.* 2014). Exposure is much more difficult to measure and to accurately ensure for observational studies than for controlled exposure studies. Therefore, exposure measurement error and misclassification are more likely to contribute to risk of bias for observational studies.

The direction of the bias (towards or away from the null) will differ based on the nature of differences between comparison groups and may be difficult to predict. Non-differential misclassification of exposure will generally bias results towards the null, but differential misclassification can bias towards or away from the null, making it difficult to predict the direction of effect (Szklo and Nieto 2007). For controlled exposure studies, noncompliance with the allocated treatment could introduce differential misclassification if compliance was unequal across study groups. Adherence to a strict study protocol that includes measures to assure or assess compliance can reduce the risk of bias.

The following methods are considered well-established and less-established:

i) Near field exposure

A well-established method for assessing RF-EMF exposure from mobile phone use is prospectively collected objective usage data (e.g. from operator data). Preferred measure is time-weighted average, or cumulative SAR value of the biologically relevant tissue as this represents the RF-EMF dose, although number of calls and duration of calls is also acceptable. Prospective self-reported mobile and cordless phone use is considered to have little bias, if unlikely to be affected by the outcome reporting. Retrospectively collected usage data has a high risk of bias. This applies to some extent also for operator recorded data as it requires accurate information on past subscriptions and many providers may delete usage data after several months.

ii) Far-field exposure

Well-established methods include whole body or organ specific SAR values, incident electric field strengths measured in V/m, magnetic fields (A/m) or power flux density (W/m²). Modelling of exposure is considered to be a direct measure of exposure, if data on the validity of the model has been presented or referenced and R^2 is (about 50% and higher) compared to a gold standard. Note that short-term measurements are not necessarily gold standard for long-term exposure assessment if one would expect high temporal variability in the measurements. Thus, the measurement period needs to be representative for the exposure period of interest. Less-established methods include geocoded distance to large transmitters, which have a higher a priori risk of bias than modelling or measurement studies.

iii) Occupational exposure

Well-established methods include direct measurements of RF-EMF exposure in the study collective. Less-established methods include the use of a job-exposure matrix from a different context.

Note: Given the fact that RF-EMF exposure situation is complex, and some misclassification is unavoidable, we will also consider the magnitude of exposure contrast as a relevant criterion in the risk of bias analysis.

Note: Consideration of Timing and Duration of Exposure: a limited duration of exposure or duration of follow-up may be problematic based on the health outcome being evaluated. However, in the context of this review and in the absence of a known mechanism, we consider any exposure duration and follow-up period of >1 week as relevant and thus not subject for risk of bias. In the evidence synthesis, we will consider exposure duration (or length of follow-up) as a modifying factor.

Note: For **far field** exposure studies focusing exclusively on mobile phone base stations, it is also required that the evaluated RF-EMF sources substantially contribute to total exposure in the study sample. Typically, near field sources are the main contributors to whole body exposure (van Wel et al, 2021) and the strength of the mobile phone base station signal is inversely correlated with the output power of mobile phones (Mazloum *et al*. 2019). This implies that in a collective of moderate to heavy mobile phone users, level of exposure from mobile phone base station is not well correlated or even negatively correlated with whole body exposure, which results in a high risk of bias for total whole-body exposure.

Definitely Low risk of bias:

Co, CaCo: There is direct evidence that exposure was consistently assessed (i.e., under the same method and time-frame) using well-established methods that directly measure exposure (e.g., measurement of the chemical in air or measurement of the chemical in blood, plasma, urine,

etc.),

OR exposure was assessed using less-established methods that directly measure exposure and are validated against well-established methods.

AND there is sufficient variation in exposure levels across groups to potentially identify associations with health outcomes (sufficient exposure contrasts refers to at least a factor of 10 (V/m, A/m or any usage measure) or 100 (W/kg, W/m²) between lowest and highest exposed groups or interquartile range)

Note: Prospective exposure assessment is a requirement for definitive low risk of bias.

Probably Low risk of bias:

Co, CaCo: There is indirect evidence that the exposure was consistently assessed using well-established methods that directly measure exposure,

OR exposure was assessed using indirect measures (e.g., questionnaire or occupational exposure assessment by a certified industrial hygienist) that have been validated or empirically shown to be consistent with methods that directly measure exposure (i.e., inter-methods validation: one method vs. another).

AND there is moderate range or variation in exposure measurements across groups to potentially identify associations with health outcomes (at least a factor of 5 (V/m, A/m or any usage measure) or 25 (W/kg, W/m²) between lowest and highest exposed groups or interquartile range)

Note: For retrospective self-reported exposure assessment, some evidence needs to be provided that recall bias did not occur.

Probably High risk of bias:

Co, CaCo: There is indirect evidence that the exposure was assessed using poorly validated methods that directly measure exposure,

OR there is direct evidence that the exposure was assessed using indirect measures that have not been validated or empirically shown to be consistent with methods that directly measure exposure (e.g., a job-exposure matrix or self-report without validation) (record “NR” as basis for answer),

OR there is insufficient information provided about the exposure assessment, including validity and reliability, but no evidence for concern about the method used (record “NR” as basis for answer).

AND there is low range or variation in exposure measurements across groups to potentially identify associations with health outcomes (a factor of 2 to 5 (V/m, A/m or any usage measure) or 4 to 25 (W/kg, W/m²) between lowest and highest exposed groups or interquartile range)

Note: No evidence provided that retrospective self-reported exposure assessment suffered from recall bias. Studies using less-established methods without validation data are of probable or definitive high risk of bias.

Definitely High risk of bias:

Co, CaCo: There is direct evidence that the exposure was assessed using methods with poor validity,

OR evidence of exposure misclassification (e.g., differential recall of self-reported exposure).

AND there is insufficient range or variation in exposure measurements across groups to potentially identify associations with health outcomes (less than a factor of 2 (V/m, A/m or any usage measure) or 4 (W/kg, W/m²) between lowest and highest exposed groups or interquartile

range)

Note: Evidence that retrospective self-reported exposure assessment suffered from recall bias.

NOTE: In a study that is considered to be of probably or definitively high risk of bias from exposure characterization, the type of exposure misclassification is also noted to inform the later evidence synthesis as the relevant bias depends on whether an association was observed or not. The following answer categories should be used:

-non-differential

-differential

-other/unclear

5. Can we be confident in the outcome assessment?

Confidence in the outcome requires valid, reliable, and sensitive methods to assess the outcome applied consistently across groups. Outcome misclassification or measurement error may be unrelated to the exposure (non-differential) or related to the exposure (differential). Non-differential measurement error of outcomes will usually bias the results toward the null by lowering precision and therefore reducing the ability to distinguish potential effects on exposure between exposure levels. Differential measurement error of outcomes can bias the exposure-outcome relationship and result in detection bias. There are three important factors for assessing bias in the outcome assessment: 1) the objectivity of the outcome assessment, 2) consistency in measurement of outcomes, and 3) blinding of the outcome assessors (for knowledge of the exposure).

Detection bias can be minimized by using valid and reliable methods to assess the outcome applied consistently across groups (i.e., under the same method and time-frame). Objectivity of the outcome assessment and the need for blinding are two sides of the same issue. Blinding requires that outcome assessors do not know the study group or exposure level of the human subject or animal when the outcome was assessed. The objectivity of procedures used for measuring and reporting an outcome will impact the degree to which outcome assessors could bias the reported results. For example, a behavioral outcome rated by a researcher (i.e., direct observation of behaviors) relies on subjective judgment and therefore may be impacted by potential bias of the outcome assessor to a greater degree than outcomes that are measured by machines (e.g., automated red blood cell counts). Similarly, studies relying on self-report of outcome may be rated as having a higher risk of bias than studies with clinically observed outcomes (Viswanathan *et al.* 2012). Although objective measures are less prone to bias by researchers than subjective measures, bias could be introduced during sample preparation or handling and therefore blinding still has a role in controlling for potential bias unless sample preparation and outcome measurement are accomplished with automated procedures. For example, the potential for outcome assessors to introduce bias would be minimized for *ex vivo* studies where samples are collected and outcomes are assessed automatically within an apparatus.

Acceptable methods for measuring the outcomes of interest will be highly dependent on the outcome and therefore a specific list of acceptable, inaccurate, or potentially biased methods should be developed for each evaluation and will require subject-matter expertise. It is recommended that experts with some knowledge of the literature (including both exposure and outcome) participate in drafting the risk-of-bias criteria for outcome assessment when a review protocol is developed. Even with early expert consultation and planning, outcome questions may arise when the actual studies are assessed because of non-traditional methods, application to non-traditional species, or endpoints that are indirectly related to the outcome of interest. Additional consultation and modifications to the outcome risk-of-bias criteria may be necessary. When changes are made, they

should be documented along with the date on which modifications were made and the logic for the changes.

Human introductory text: Differential methods used in the assessment of outcomes is a source of bias and this is a widely used risk-of-bias element in tools for observational human studies (Downs and Black 1998, Genaidy *et al.* 2007, Shamliyan *et al.* 2010, Viswanathan *et al.* 2012, Sterne *et al.* 2014). The recent guidance for non-randomized studies of interventions suggests considering the objectivity of the outcome assessment when evaluating bias in the outcome assessment (Sterne *et al.* 2014) and we have included consideration of the objectivity in this document for evaluating the potential impact of blinding practices. Blinding of outcome assessors is a widely recommended risk-of-bias element for controlled trials and observational studies (Higgins and Green 2011, Viswanathan *et al.* 2012, Sterne *et al.* 2014). For human studies blinding of the subject to exposure levels should also be considered. For example, a subject's knowledge of their own exposure levels would represent an increased risk of bias for self-reported outcomes relative to clinically measured outcomes.

Without distinguishing between the different stages of blinding during the conduct of a study, lack of blinding in randomized trials has been empirically shown to be associated with larger estimations of intervention effects (on average a 9% increase in an odds ratio) (Pildal *et al.* 2007). Schulz et al. (1995) analyzed 250 controlled trials and found that studies that were not double-blinded had a 17% larger estimation of treatment effect, on average. In trials with more subjective outcomes, more bias has been observed with lack of blinding (Wood *et al.* 2008), indicating that blinding outcome assessors could be more important for these effects.

For some exposures, it is not possible to entirely blind outcome assessors, particularly if subjects are self-reporting outcomes. In practice, successful blinding cannot always be ensured, as it can be compromised for most interventions. In some cases the treatment may have side effects possibly allowing the participant to detect which intervention they received, unless the study compares interventions with similar side effects or uses an active placebo (Boutron *et al.* 2006).

The nature of these outcomes means that primarily self-reported symptoms, including any bodily sensation or a feeling or change in well-being, are obtained by a written questionnaire or personal interview. We will consider as 'reliable' the use of methods, scales, scores, which authors referenced as having been validated and sensitive to measure long term effects. It is acceptable that for the outcomes (e.g. headache, sleep quality) no other gold standard than self-reported exists. However, compared to self-administered questionnaire in a research setting, higher validity is given if symptoms have been reported to a health professional in the framework of an anamnesis. It is also crucial that outcome reporting and the instrument used for it refers to a biologically plausible time window with respect to the exposure of interest.

A particular challenge in this field is that both, outcome and exposure (e.g. duration of mobile phone use), may be self-reported. By definition, participants are thus aware of their exposure status and this may affect their outcome reporting. However, researchers may have taken several measures to minimize such bias, e.g. in the framing of the study and by not focusing on one specific type of exposure. Such measures may prevent blinding bias to some extent. Another situation with minimal bias would be use of medical records established by general practitioners without a direct link to the study objective.

Definitely Low risk of bias:

Co: There is direct evidence that the outcome was assessed using well-established methods (e.g., the "gold standard" with validity and reliability >0.70 (Genaidy *et al.* 2007)),

AND subjects had been followed for the same length of time in all study groups. Acceptable assessment methods will depend on the outcome, but examples of such methods may include: objectively measured with diagnostic methods, measured by trained interviewers, obtained from registries (Shamliyan *et al.* 2010),

AND there is direct evidence that the outcome assessors (including study subjects, if outcomes were self-reported) were adequately blinded to the study group, and it is unlikely that they could have broken the blinding prior to reporting outcomes.

CaCo: There is direct evidence that the outcome was assessed in cases (i.e., case definition) and controls using well-established methods (the gold standard),

AND subjects had been followed for the same length of time in all study groups,

AND there is direct evidence that the outcome assessors (including study subjects, if outcomes were self-reported) were adequately blinded to the exposure level when outcome was assessed in cases (i.e., case definition) and controls.

Note: By definition, study subjects are not blind to exposure surrogates like mobile phone use or occupational situations. These studies should not receive definitive low risk of bias. However, study participants are unlikely to be aware of their exposure status from fixed site transmitters.

Probably Low risk of bias:

Co: There is indirect evidence that the outcome was assessed using acceptable methods (i.e., deemed valid and reliable but not the gold standard) (e.g., validity and reliability ≥0.40 (Genaidy *et al.* 2007)),

AND subjects had been followed for the same length of time in all study groups [Acceptable, but not ideal assessment methods will depend on the outcome, but examples of such methods may include proxy reporting of outcomes and mining of data collected for other purposes],

OR it is deemed that the outcome assessment methods used would not appreciably bias results,

AND there is indirect evidence that the outcome assessors (including study subjects, if outcomes were self-reported) were adequately blinded to the study group, and it is unlikely that they could have broken the blinding prior to reporting outcomes,

OR it is deemed that lack of adequate blinding of outcome assessors would not appreciably bias results, which is more likely to apply to objective outcome measures.

CaCo: There is indirect evidence that the outcome was assessed using acceptable methods (i.e., deemed valid and reliable but not the gold standard) (e.g., validity and reliability ≥0.40 (Genaidy *et al.* 2007)),

AND subjects had been followed for the same length of time in all study groups [Acceptable, but not ideal assessment methods will depend on the outcome, but examples of such methods may include proxy reporting of outcomes and mining of data collected for other purposes],

OR it is deemed that the outcome assessment methods used would not appreciably bias results,

AND there is indirect evidence that the outcome assessors (including study subjects, if outcomes were self-reported) were adequately blinded to the study group, and it is unlikely that they could have broken the blinding prior to reporting outcomes,

OR it is deemed that lack of adequate blinding of outcome assessors would not appreciably bias results, which is more likely to apply to objective outcome measures.

Note: Although, by definition study subjects are not blind to exposure surrogates like mobile phone use, a study may be considered of low risk of bias, if convincing measures have been taken to minimize bias on outcome reporting.

Probably High risk of bias:

Co: There is indirect evidence that the outcome assessment method is an insensitive instrument (e.g., a questionnaire used to assess outcomes with no information on validation),

OR the length of follow up differed by study group,

OR there is indirect evidence that it was possible for outcome assessors (including study subjects if outcomes were self-reported) to infer the study group prior to reporting outcomes,

OR there is insufficient information provided about blinding of outcome assessors (record "NR" as basis for answer).

CaCo: There is indirect evidence that the outcome was assessed in cases (i.e., case definition) using an insensitive instrument,

OR there is insufficient information provided about how cases were identified (record "NR" as basis for answer),

OR there is indirect evidence that it was possible for outcome assessors to infer the exposure level prior to reporting outcomes (including that subjects self-reporting outcomes were likely aware of reported links between the exposure and outcome),

OR there is insufficient information provided about blinding of outcome assessors (record "NR" as basis for answer).

Definitely High risk of bias:

Co: There is direct evidence that the outcome assessment method is an insensitive instrument,

OR the length of follow up differed by study group,

OR there is direct evidence for lack of adequate blinding of outcome assessors (including study subjects if outcomes were self-reported), including no blinding or incomplete blinding.

CaCo: There is direct evidence that the outcome was assessed in cases (i.e., case definition) using an insensitive instrument,

OR there is direct evidence that outcome assessors were aware of the exposure level prior to reporting outcomes (including that subjects self-reporting outcomes were aware of reported links between the exposure and outcome).

(E) Selective Reporting Bias (ALL)

Selective reporting bias refers to selective inclusion of outcomes in the publication of the study based on the results (Hutton and Williamson 2000, Higgins and Green 2011).

6. Were all measured outcomes reported?

Selective reporting of results is a recommended element of assessing risk of bias (Guyatt *et al.* 2011, Higgins *et al.* 2011, IOM 2011, Viswanathan *et al.* 2012). Selective reporting is present if pre-specified outcomes are not reported or incompletely reported. It is likely widespread and difficult to assess with confidence for most studies unless the study protocol is available. Selective reporting bias can be assessed by comparing the "methods" and "results" section of the paper, and by considering outcomes measured in the context of knowledge in the field. Abstracts of presentations relating to the study may contain information about outcomes not subsequently mentioned in publications. Selective reporting bias should be suspected if

the study does not report outcomes in the results section that would have been expected based on the methods, or if a composite score is present without the individual component outcomes (Guyatt *et al.* 2011). It may be useful to pay attention to author affiliations and funding source which can contribute to selective outcome reporting when results are not consistent with expectations or value to the research objectives.

For this item it is also required to evaluate whether exposure-response association was a priori defined or whether this is data driven. Direct evidence is obtained from a pre-published study protocol or analysis plan. In the absence of direct evidence, use of uncommon exposure categorization cut-offs may be an indication for data driven approaches (instead of quantiles or absolute values used in previous studies). It may also be useful to check various publications from the same study, whether the same analysis strategies have been used. If not, it should be checked whether there are plausible arguments for varying approaches.

Definitely Low risk of bias:

Co, CaCo: There is direct evidence that all of the study's measured outcomes (primary and secondary) outlined in the protocol, methods, abstract, and/or introduction (that are relevant for the evaluation) have been reported. This would include outcomes reported with sufficient detail to be included in meta-analysis or fully tabulated during data extraction and analyses had been planned in advance.

Probably Low risk of bias:

Co, CaCo: There is indirect evidence that all of the study's measured outcomes (primary and secondary) outlined in the protocol, methods, abstract, and/or introduction (that are relevant for the evaluation) have been reported,

OR analyses that had not been planned in advance (i.e., retrospective unplanned subgroup analyses) are clearly indicated as such and it is deemed that the unplanned analyses were appropriate and selective reporting would not appreciably bias results (e.g., appropriate analyses of an unexpected effect). This would include outcomes reported with insufficient detail such as only reporting that results were statistically significant (or not).

Probably High risk of bias:

Co, CaCo: There is indirect evidence that all of the study's measured outcomes (primary and secondary) outlined in the protocol, methods, abstract, and/or introduction (that are relevant for the evaluation) have been reported,

OR and there is indirect evidence that unplanned analyses were included that may appreciably bias results,

OR there is insufficient information provided about selective outcome reporting (record "NR" as basis for answer).

Definitely High risk of bias:

Co, CaCo: There is direct evidence that all of the study's measured outcomes (primary and secondary) outlined in the protocol, methods, abstract, and/or introduction (that are relevant for the evaluation) have not been reported. In addition to not reporting outcomes, this would include reporting outcomes based on composite score without individual outcome components or outcomes reported using measurements, analysis methods or subsets of the data (e.g., subscales) that were not pre-specified or reporting outcomes not pre-specified, or that unplanned analyses were included that would appreciably bias results.

(F) Other Bias

7. Were there no other potential threats to internal validity (e.g., statistical methods were appropriate and researchers adhered to the study protocol)?

On a project specific basis, additional questions for other potential threats to internal validity can be added and applied to study designs as appropriate. Note that we do not consider one question proposed by OHAT tool under this domain for case-control and cohort studies, which is adherence to the study protocol. Study protocols are rarely written in this specific field of observational studies. Also OHAT stated in their instruction: "The overwhelming majority of studies examined during case study evaluations were not reported in sufficient detail to permit a meaningful answer to whether or not the study adhered to a study protocol." Thus, this dimension is not used.

7a. Were statistical methods appropriate?

Some of the more extensive quality tools have a separate question for appropriateness of the statistical methods (e.g., 1 of the 25 elements in the Downs and Black 1998 tool addresses the statistics); however most do not include a separate question. The OHAT risk-of-bias tool suggests consideration of statistical methods with the other potential threats to internal validity. One of the common statistical issues identified has been reporting of statistical tests that require normally distributed data (e.g., t-test or ANOVA) without reporting that the homogeneity of variance was tested or confirmed.

It is recommended that experts with some knowledge of statistical methods used in the literature participate in drafting the risk-of-bias criteria for identifying inappropriate statistical methods when a review protocol is developed. Even with early expert consultation and planning, statistical methods questions may arise when the actual studies are assessed. Additional consultation and modifications to the statistical methods risk-of-bias criteria may be necessary. When changes are made, they should be documented along with the date on which modifications were made and the logic for the changes.

Symptom score data may rarely follow a normal distribution but rather a negative binomial or Poisson distribution. It should thus be checked whether adequate statistical methods have been used or any selection has been justified.

7b. Is there evidence for reverse causality?

For instance, it is well established that IEI-EMF individuals take measures to reduce their RF-EMF exposure when developing symptoms. If not adequately considered in the longitudinal design, this would downward bias the effect estimates because occurrence of symptoms is negatively correlated with exposure status. This would produce a bias towards a false protective effect of RF-EMF. Conversely, it is conceivable that e-media use is related to latent variables, which are causing reverse causality. For instance, degree of hyperactivity in children, although not diagnosed and not pathological, may predict future e-media use.

Another concern for reverse causality are analysis approaches based on cumulative exposure or changes in exposure between baseline and follow-up. Such an analysis is justified from a biological point of view. However, there is a risk that development of outcome in an early state of the follow-up period may have affected the exposure situation. For example, a person suffering from sleeping problems may increase nighttime mobile phone use, which would create a spurious association between RF-EMF exposure and symptom occurrence (false positive risk). The likelihood for such a potential bias should be assessed for the specific PECO question under consideration. Depending on the severity of the outcome such type of bias may be

judged to be implausible for far field and occupational exposure.

Definitely Low risk of bias:

Co, CaCo: There is direct evidence that the study population has not taken any measure to reduce their RF-EMF exposure because they suspected own symptoms to be related to the exposure of interest.

Probably Low risk of bias:

Co, CaCo: There is indirect evidence that the study population has not taken any measure to reduce their exposure because they suspected own symptoms to be related to the EMF exposure.

Probably High risk of bias:

Co, CaCo: There is indirect evidence that a relevant part (ca. >10%) of the study population has taken measures to reduce their exposure because they suspected own symptoms to be related to the EMF exposure.

Definitely High risk of bias:

Co, CaCo: There is direct evidence that a relevant part of the study population has taken measures to reduce their exposure because they suspected own symptoms to be related to the EMF exposure.

NTP. OHAT: Risk of Bias Rating Tool for Human and Animal Studies. US NIEHS NIH: National Toxicology Program, Office of Health Assessment and Translation (OHAT); 2015
