# Supplementary material for: The effects of radiofrequency electromagnetic fields exposure on tinnitus, migraine and non-specific symptoms in the general and working population: A protocol for a systematic review on human observational studies
Source: Environ Int. 2021 Dec;157:106852. doi: 10.1016/j.envint.2021.106852 (PMC8484767; doi:10.1016/j.envint.2021.106852)
Supplement: Supplementary data 4 [file mmc4.pdf]

Appendix D: Template Summary of the evidence rating

Instruction: Fill in the table and use the footnotes for the rationales. Text in brackets must be adapted or used if relevant for the judgement. For downgrading or upgrading additional arguments should be reported than provided in the template.

Table 1: Summary of the evidence-rating scheme for each set of PECO

**Type of Population**				
**Type of Exposure**				
**Type of Comparator**				
**Type of Outcome**				
**Summary of findings**	**Effect estimates in relation to exposure of interest**		*	
	**Number of participants (# evaluated studies)**			
	**Number of cases**			
			**Rating**	**Adjustment to rating**
**Quality assessment**	**Starting rating**		a	
	**Factors decreasing confidence**	**Risk of bias**	b	
		**Inconsistency**	**c**	
		**Indirectness**	**d**	
		**Imprecision**	**e**	
		**Publication bias**	**f**	
	**Factors increasing confidence**	**Strength of association**	g	
		**Exposure-response gradient**	h	
		**Residual confounding**	i	
	**Overall judgement of the quality of evidence**			

* The estimate is based on ___ effect estimates;

a We started with a grading of "____" (__), because…

b Risk of bias was judged to be (not likely / serious / very serious), because… (Some studies on local exposure found an association with the outcome and in-depth analysis suggests that the association was rather related to usage than due to RF-EMF exposure.)

c Inconsistency was rated to be (none /serious). The number of studies was (sufficient/insufficient) to do a heterogeneity analysis. (We found (I^2)… The predictive interval (included/excluded) 1). This implies that we (did not find / found serious) heterogeneity between studies.

d Indirectness was considered (not relevant / serious) because the evaluated studies (did not assess / assessed) population, exposure, and outcome of interest. (In particular…)

e We considered the results to be (precise / imprecise) because (the upper limit of the confidence interval was found to be _____ for a non-significant effect estimate / the upper limit of the confidence estimate divided by the point estimate was_____ for a significant effect estimate.

f There was (no) reason to believe that there is some publication bias or small study bias because (Egger test, funding, unpublished abstracts, early unconfirmed positive findings).

g Effect was found to be (small / large / very large). The relative risk was ____ per Interquartile increase in exposure (____)

h Exposure-response gradient was (not) found to be monotonic based (on a test for trend).

Appendix D: Template Summary of the evidence rating

i We (did not find / found) evidence to suggest that possible residual confounders or biases had reduced the observed effect estimate. (Some studies on local exposure found an association with the outcome and in-depth analysis suggests that the association was rather related to RF-EMF exposure than due to usage.)
